# A phage-encoded RNA-binding protein inhibits the antiviral activity of a toxin–antitoxin system

**DOI:** 10.1093/nar/gkad1207

**Published:** 2023-12-20

**Authors:** Chantal K Guegler, Gabriella I C Teodoro, Sriram Srikant, Keerthana Chetlapalli, Christopher R Doering, Dia A Ghose, Michael T Laub

**Affiliations:** Department of Biology, Massachusetts Institute of Technology, Cambridge, MA 02139, USA; Department of Genetics, Harvard Medical School, Boston, MA 02115, USA; Department of Biology, Massachusetts Institute of Technology, Cambridge, MA 02139, USA; Department of Biology, Massachusetts Institute of Technology, Cambridge, MA 02139, USA; Department of Biology, Massachusetts Institute of Technology, Cambridge, MA 02139, USA; Department of Biology, Massachusetts Institute of Technology, Cambridge, MA 02139, USA; Department of Biology, Massachusetts Institute of Technology, Cambridge, MA 02139, USA; Department of Biology, Massachusetts Institute of Technology, Cambridge, MA 02139, USA; Howard Hughes Medical Institute, Massachusetts Institute of Technology, Cambridge, MA 02139, USA

## Abstract

Bacteria harbor diverse mechanisms to defend themselves against their viral predators, bacteriophages. In response, phages can evolve counter-defense systems, most of which are poorly understood. In T4-like phages, the gene *tifA* prevents bacterial defense by the type III toxin–antitoxin (TA) system *toxIN*, but the mechanism by which TifA inhibits ToxIN remains unclear. Here, we show that TifA directly binds both the endoribonuclease ToxN and RNA, leading to the formation of a high molecular weight ribonucleoprotein complex in which ToxN is inhibited. The RNA binding activity of TifA is necessary for its interaction with and inhibition of ToxN. Thus, we propose that TifA inhibits ToxN during phage infection by trapping ToxN on cellular RNA, particularly the abundant 16S rRNA, thereby preventing cleavage of phage transcripts. Taken together, our results reveal a novel mechanism underlying inhibition of a phage-defensive RNase toxin by a small, phage-encoded protein.

## Graphical Abstract

**Graphical Abstract:**
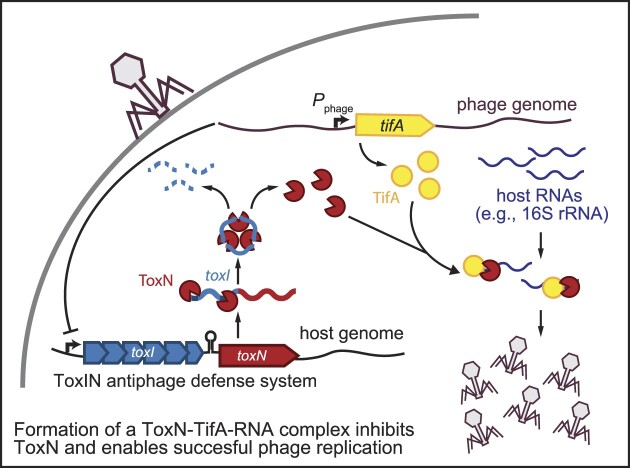

## Introduction

Predation by phages, the viruses that infect bacteria, represents a constant and potent threat to bacterial life. The need to survive this conflict has driven the evolution of numerous highly sophisticated antiphage elements in bacteria, including restriction-modification and CRISPR-Cas systems (1–3). These mechanisms, in turn, have led to the emergence of counter-defense systems in phages, highlighting the intense, ongoing evolutionary arms race between phages and their bacterial hosts (4,5). In addition to illuminating the fundamental mechanisms underlying these host-pathogen interactions, understanding this arms race is crucial for the development of phage therapy to combat the rise of antibiotic-resistant bacteria.

One prevalent class of bacterial defense genes is toxin–antitoxin (TA) systems, widespread genetic elements comprising a growth-inhibitory toxin that is neutralized by its cognate antitoxin (6–16). During phage infection, the toxin is liberated (‘activated’) from the antitoxin and goes on to block phage replication (9). Most toxins are growth inhibitory rather than bactericidal, but many TA systems have been proposed to act via an abortive infection mechanism. In such a mechanism, the infected cell does not survive phage infection (17,18). However, by blocking phage propagation, the TA system prevents spread of the phage from infected cells to the rest of a bacterial population (11,13,14,19).

TA systems are grouped into ‘types’ based on the molecular mechanism underlying antitoxin inhibition of the cognate toxin (6,8). The type III TA systems, first discovered in the plant pathogen *Pectobacterium atrosepticum*, typically involve an endoribonuclease (RNase) toxin and an antitoxin RNA expressed as an array of short tandem repeats (ToxN and *toxI*, respectively, in *P. atrosepticum*) (11,20–22). The toxin cleaves these repetitive antitoxin RNAs into shorter repeat monomers (typically 36–50 nucleotides in length); the interactions between the toxin and antitoxin neutralize the toxin by sequestering it from other putative substrate RNAs in the cell (20,23,24). Following infection, phage-induced shutoff of host transcription leads to rapid depletion of the cellular pool of unstable antitoxin RNAs, thereby liberating the toxin (19). Activated toxin then cleaves phage mRNAs to block production of new phage particles.

How do phages overcome or escape from the defense mediated by type III TA systems? In *P. atrosepticum*, the phage ΦTE can overcome *toxIN* by amplifying a locus expressing a noncoding RNA that resembles *toxI* or by acquiring *toxI* from the *toxIN* system directly (25). Further, the *P. atrosepticum* phage ΦM1 can acquire single-base changes in a gene of unknown function called M1-23, which interacts with the host nucleotide excision repair protein UvrA and allows ΦM1 to evade *toxIN* and another type III TA system called *tenpIN* (26). A T4-like phage that infects *Serratia* overcomes *toxIN* through mutations in the phage gene *asiA*, which encodes a factor that helps reprogram the host RNA polymerase to transcribe phage genes (27). The *asiA* mutation likely prevents full transcriptional shutoff of *toxIN*, which is required for ToxN activation.

**Figure 1.**
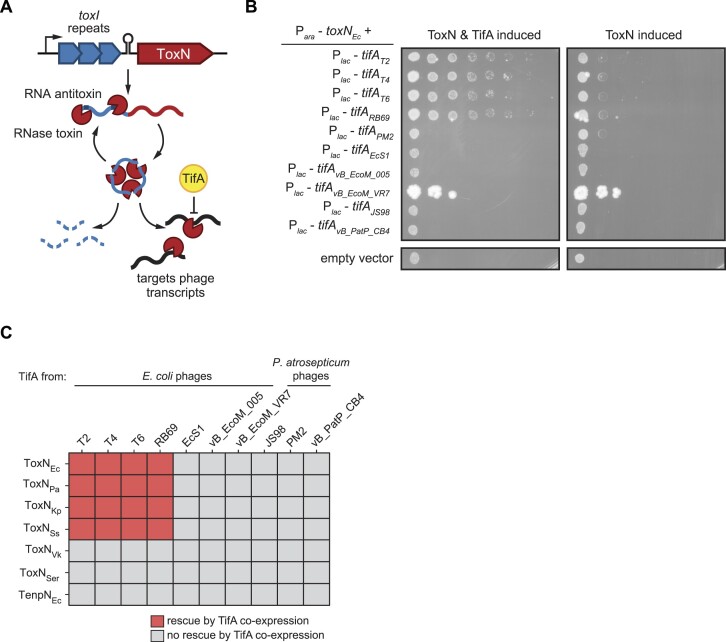
TifA is a small phage-encoded inhibitor of ToxN. (**A**) Model for ToxIN complex formation, activation, and inhibition by TifA during phage infection. (**B**) Serial dilutions of *E. coli* cells expressing *toxN_Ec_* and several *tifA* homologs. Plasmids harboring *toxN* and *tifA* under arabinose- and IPTG-inducible promoters, respectively, were transformed into *E. coli* MG1655, and expression was induced as indicated with the addition of arabinose and IPTG. (**C**) Heatmap showing the ability of various TifA homologs to rescue overproduction of various ToxN homologs; for plate images, see Supplementary Figure S2A-F. Bacterial species from which ToxN and TenpN homologs were cloned: *Escherichia coli* (Ec); *Pectobacterium atrosepticum* (Pa); *Klebsiella pneumoniae* (Kp); *Shigella sonnei* (Ss); *Vibrio kanaloae* (Vk); *Serratia* sp. SRS-8-S-2018 (Ser). Also see Supplementary Figures S1 and S2.

We recently described a *toxIN* homolog from an environmental isolate of *Escherichia coli* that provides *E. coli* MG1655 with robust defense against several phages, including T4, T5, and T7 (19). We then evolved T4 to escape *toxIN*-mediated defense. Sequencing demonstrated that phages overcame *toxIN* via segmental amplification, and thus increased expression, of the T4 early gene *tifA* (previously *61.4*), which encodes a protein inhibitor of ToxN (28). TifA is a small (85 amino acid) protein that is conserved across T-even phages. Although ToxN co-precipitates with TifA during T4 infection, the mechanistic basis of TifA inhibition of ToxN remains unclear (28) (Figure 1A).

Here, we show that TifA proteins from different T-even phages are specific inhibitors of several ToxN homologs, indicating that TifA proteins recognize certain features of ToxN and are not nonspecific inhibitors of RNase activity. Using structure predictions coupled to a genetic screen, we identify the regions of TifA that likely bind ToxN, including one extended loop that may partially occlude the ToxN active site. Notably, we also show that TifA is a nucleic acid-binding protein and that TifA together with ToxN forms a high molecular weight RNA-protein complex. RNA-sequencing indicates that 16S rRNA is highly enriched in the complexes formed. We propose that TifA inhibits ToxN during phage infection by tethering it to 16S rRNAs, and possibly other cellular RNAs, thereby rendering it inert. Taken together, our results uncover a novel mechanism underlying inhibition of an RNase toxin by a phage-encoded antitoxin and highlight the role that type III TA systems play in the phage-bacterial arms race.

## Materials and methods

### Reagents, software and databases

All commercial instruments, reagents and software used in this study are listed in Supplementary Table S1.

### Biological resources

All strains, plasmids, and primers used in this study are listed in Supplementary Tables S3, S4, and S5, respectively. Phage Thu1/R2 was isolated during the 2021 Fall LS50 course (Harvard) by freshman students under the instruction of Sriram Srikant. It was isolated from Charles River water on *E. coli* MG1655, and genome sequenced to taxonomically classify it in the *Tevenvirinae*.

### Strains and growth conditions

For all cell spotting experiments, *E. coli* MG1655 strains were grown in M9 medium (10x stock made with 64 g/l Na_2_HPO_4_-7H_2_O, 15 g/l KH_2_PO_4_, 2.5 g/l NaCl, 5.0 g/l NH_4_Cl) supplemented with 0.4% glycerol, 0.1% casamino acids, 2 mM MgSO_4_, and 0.1 mM CaCl_2_ (M9-glycerol). When applicable, 0.4% glucose was supplemented to prevent leaky toxin expression from the arabinose-inducible P*_BAD_* promoter (M9-glucose). For plasmid construction, *E. coli* TOP10 or DH5α cells were cultured in Luria broth (LB) medium, supplemented with 0.4% glucose when applicable. For phage infection experiments, *E. coli* MG1655 strains were grown in LB medium. For protein overexpression, *E. coli* T7 express cells (NEB) were grown in LB medium. Antibiotics were used at the following concentrations (liquid; plates): carbenicillin (50 μg/ml; 100 μg/ml), chloramphenicol (20 μg/ml; 30 μg/ml).

### Plasmid construction

pBAD33-*toxN* homologs: ToxN homolog sequences screened in Figure 1 were ordered in G-blocks from IDT, which also contained flanking SalI and SacI sites (Supplementary Table S6). The G-blocks were then cloned into the SalI and SacI sites of the arabinose-inducible pBAD33 vector (pBAD33-*toxN*). To construct pBAD33-*toxN_Ec_*-CBD (chitin binding domain), *toxN* fused to the Mxe GyrA intein and the chitin binding domain was PCR-amplified from pTXB1-*toxN* with primers CKG-661/662, which also introduced complementarity to the pBAD33 backbone. pBAD33 was linearized with SalI-HF and SacI-HF, and the PCR products were ligated using Gibson assembly.

pEXT20-*tifA* homologs: TifA homolog sequences screened in Figure 1 were ordered in G-blocks from IDT (Supplementary Table S6) or amplified from phage genomic DNA and cloned into pKVS45 using Gibson assembly. The TifA homologs were then PCR-amplified from pKVS45 using primers listed in Supplementary Table S5 and ligated into SacI/SalI-linearized pEXT20 vector using Gibson assembly. To test for expression of TifA homologs in *E. coli*, pEXT20-*tifA_vB_PatP_CB4_*-FLAG and pEXT20-*tifA_vB_EcoM_005_*-FLAG were constructed using around-the-horn PCR of pEXT20-*tifA_vB_PatP_CB4_* and pEXT20-*tifA_vB_EcoM_005_* using primer pairs CKG-666/672 and CKG-666/667, respectively.

pBR322-*toxIN* and pBR322-*toxI-toxN*-His_6_ mutants: pBR322-*toxI-toxN(Y115H)*, pBR322-*toxI-toxN(L118S)*, pBR322-*toxI-toxN(K111N)*, and pBR322-*toxI-toxN(K111R)* were constructed using around-the-horn PCR of pBR322-*toxIN* using primer pairs CKG-499/500, CKG-501/502, CKG-545/546 and CKG-547/546, respectively. pBR322-*toxI-toxN(K55A)*-His_6_ was constructed using around-the-horn PCR of pBR322-*toxI-toxN(K55A)* with the primer pair CKG-303/304.

pEXT20-*tifA_T4_* and pEXT20-*tifA_RB69_* mutants: pEXT20-*tifA_T4_(F41S)* and pEXT20-*tifA_T4_(F41H)* were constructed using around-the-horn PCR of pEXT20-*tifA_T4_* with primer pairs CKG-597/598 and CKG-599/598, respectively. pEXT20-*tifA_RB69_(H41F)* and pEXT20-*tifA_RB69_(H41S)* were constructed using around-the-horn PCR of pEXT20-*tifA_RB69_* with primer pairs CKG-600/601 and CKG-602/601, respectively. pEXT20-*tifA_RB69_(P53A T54A)* and pEXT20-*tifA_RB69_(R52A K55A R56A R59A)* were constructed by Gibson assembly of G-blocks (Supplementary Table S6) into pEXT20 vector linearized via digestion with SacI/SalI. pEXT20-*tifA_RB69_(K55A R56A R59A)* was then constructed from pEXT20-*tifA_RB69_(R52A K55A R56A R59A)* via around-the-horn PCR with primers CKG-625/626. pEXT20-*tifA_RB69_*-FLAG was constructed via around-the-horn PCR of pEXT20-*tifA_RB69_* with primers CKG-665/441. To construct pEXT20-*tifA_RB69_*-MBP-His_6_, MBP-His_6_ was PCR-amplified from pET-*tifA_RB69_*-MBP-His_6_ with primers CKG-663/664. pEXT20-*tifA_RB69_* was linearized via PCR with CKG-624/665. The PCR products were then ligated together using Gibson assembly.

pKVS45-*tifA_T4_*-FLAG mutants: pKVS45-*tifA_T4_(F41S)*-FLAG, pKVS45-*tifA_T4_(F41H)*-FLAG, and pKVS45-*tifA_T4_(P53A T54A)*-FLAG were constructed via around-the-horn PCR of pKVS45-*tifA_T4_*-FLAG using primer pairs CKG-597/598, CKG-599/598, and CKG-603/604, respectively. pKVS45-*tifA_T4_(R52A K55A R56A R59A)* was constructed by around-the-horn PCR of pKVS45-*tifA_T4_* using primers CKG-523/524. A FLAG tag was then added to pKVS45-*tifA_T4_(R52A K55A R56A R59A)* with around-the-horn PCR using primers CKG-411/404. pKVS45-*tifA_T4_(K55A R56A R59A)*-FLAG was then constructed from pKVS45-*tifA_T4_(R52A K55A R56A R59A)*-FLAG via around-the-horn PCR with CKG-627/628.

pTXB1-*toxN*: The *toxN* gene was PCR-amplified from pBAD33-*toxN* using primers CKG-413/415, which also introduced an extra arginine residue to the C-terminus of ToxN (to increase intein self-cleavage efficiency) and NdeI and SapI restriction sites. The PCR product was then cloned into the NdeI and SapI sites of the protein overexpression vector pTXB1 (NEB), upstream of a C-terminal intein and chitin-binding domain.

pACYC-*toxI*: *toxI* was PCR-amplified, along with its native promoter and transcription terminator, from pBR322-*toxIN* using CKG-416/139. pACYCDuet-1 (Novagen) was linearized via PCR with CKG-147/134 to remove the first RBS and the second T7 promoter and RBS from the vector. *toxI* was then ligated into linearized pACYC with Gibson assembly.

pACYC-*tifA_RB69_* and pACYC-*tifA_RB69_*-FLAG: *tifA_RB69_* was PCR-amplified from pKVS45-*tifA_RB69_* with using CKG-450/451. pACYCDuet-1 was linearized via PCR with CKG-423/424 to remove the second T7 promoter and RBS from the vector. *tifA_RB69_* was then ligated into linearized pACYC with Gibson assembly. pACYC-*tifA_RB69_*-FLAG was then constructed from around-the-horn PCR of pACYC-*tifA_RB69_* with CKG-441/424.

pACYCDuet-1-*toxI*-*tifA_RB69_*, pACYCDuet-1-*toxI*-*tifA_RB69_-*FLAG and pACYCDuet-1-*toxI*-*tifA_RB69_(K55A R56A R59A)*: *toxI* was PCR-amplified, along with its native promoter and transcription terminator, from pACYC-*toxI* using CKG-416/460. pACYCDuet-1 was linearized via PCR with CKG-147/444, and *toxI* was ligated downstream of the first T7 promoter into linearized pACYCDuet-1 with Gibson assembly to yield pACYCDuet-1-*toxI*. *tifA_RB69_* was PCR-amplified from pKVS45-*tifA_RB69_* with CKG-458/459, which also introduced flanking AvrII and NdeI restriction sites. The PCR product was then cloned into the AvrII and NdeI sites of pACYCDuet-1-*toxI* downstream of the second T7 and RBS promoter to yield pACYCDuet-1-*toxI*-*tifA_RB69_*. pACYCDuet-1-*toxI*-*tifA_RB69_*-FLAG was then constructed from around-the-horn PCR of pACYC-*tifA_RB69_* with CKG-441/575. pACYCDuet-1-*toxI*-*tifA_RB69_ (K55A R56A R59A)* was constructed via around-the-horn PCR of pACYC-*tifA_RB69_* with CKG-656/657.

pET-*tifA_RB69_*-MBP-His_6_: *tifA_RB69_* was introduced into pET-MBP-His_6_ (Addgene Plasmid #37237) using ligation-independent cloning. *tifA_RB69_* was PCR-amplified from pACYC-*tifA_RB69_* with PCR primers CKG-465/466, gel-purified, and digested with T4 DNA polymerase in the presence of dGTP to create sticky ends with homology to pET-MBP-His_6_. pET-MBP-His_6_ was linearized via restriction digestion with HpaI and then and digested with T4 DNA polymerase in the presence of dCTP to create sticky ends. The insert and vector were then incubated together at room temperature for 15 min. 1 μl 25 mM EDTA was then added, and the ligation was incubated for an additional 30 min at room temperature to yield pET-*tifA_RB69_*-MBP-His_6_. pET-*tifA_RB69_(P53A T54A)*-MBP-His_6_ and pET-*tifA_RB69_(K55A R56A R59A)*-MBP-His_6_ were assembled analogously; *tifA_RB69_(P53A T54A)* was PCR-amplified from pEXT20-*tifA_RB69_(P53A T54A)* with primers CKG-465/466, and *tifA_RB69_(K55A R56A R59A)* was PCR-amplified from pEXT20-*tifA_RB69_(K55A R56A R59A)* with primers CKG-465/466.

### ToxN, *toxI* and TifA overproduction on solid media

ToxN and TifA overproduction spotting assays were conducted as described previously (19,28). Briefly, single colonies of each strain were grown to saturation overnight at 37°C in M9-glucose. 1 ml of each culture was then harvested by centrifugation, washed twice in 1× PBS, and resuspended in 500 μl 1× PBS. Resuspended cells were serially diluted 10-fold in 1× PBS and spotted on M9L plates (M9-glycerol supplemented with 5% (v/v) LB) supplemented with 0.2% arabinose (toxin-inducing), 0.2% arabinose and 100 μM IPTG (toxin- and TifA/*toxI*-inducing), and 0.4% glucose (toxin-suppressing). Plates were then incubated at 37°C for 24 h (M9L-glucose) or 48 h (M9L-arabinose and M9L-arabinose-IPTG) before imaging. Images shown in figures are representative of at least three independent biological replicates.

### Western blotting to measure expression levels of *tifA* homologs

To verify expression of selected *tifA* homologs, pEXT20-*tifA_vB_PatP_CB4_*-FLAG and pEXT20-*tifA_vB_EcoM_005_*-FLAG were transformed into *E. coli* MG1655. Overnight cultures containing each plasmid were back-diluted 1:100 and grown to mid-log phase in a shaking water bath at 37°C in M9-glycerol supplemented with 100 μM IPTG to induce *tifA* expression. Cells were harvested ∼6.5 h after dilution via centrifugation, and protein expression was analyzed with western blotting using an anti-FLAG antibody (Cell Signaling Technologies) as described previously (28). RpoA was used as a loading control by probing membranes with an anti-*E. coli* RpoA antibody (BioLegend).

### *tab* screen

The *toxN* gene was mutagenized using error-prone PCR following the protocol described in (29). Briefly, *toxN* was PCR-amplified with primers CKG-106/12 from pBR322-*toxIN* in 1x Taq reaction buffer using Taq polymerase in the presence of 0.125 mM and 0.0625 mM MnCl_2_. The PCR products were then gel-purified and re-introduced into pBR322-*toxI* backbone (linearized with CKG-497/498) using Gibson assembly. Prior to Gibson assembly, the mutagenized *toxN* PCR products were also TOPO cloned and Sanger sequenced to ensure that there were 0–1 mutations per gene present.

2 μl of each Gibson-assembled pBR322-*toxIN* plasmid was then transformed into *E. coli* TOP10, grown overnight at 37°C in 5 ml LB + carb, and miniprepped. Miniprepped libraries, alongside wild-type pBR322-*toxIN*, were then transformed using electroporation into MG1655 pKVS45-*tifA*_T4_ and MG1655 pKVS45-*tifA*_RB69_ and grown overnight at 37°C in 5 ml LB + carb + chlor.

The *tab* screen was then conducted as described in (16). Briefly, overnight cultures were back-diluted to OD_600_ = 1.0 in LB + carb. 100 μl of each culture was mixed separately with 100 μl of a range of T4 dilutions (5.5 × 10^6^ to 5.5 × 10^10^ pfu/ml) and 20 ml of molten LB + 0.5% agar and spread to cover the bottom of an empty 15 cm Petri dish. Plates were incubated overnight at 37°C to allow microcolonies to form. Colonies from plates containing the phage dilution with the largest difference in colony-forming efficiency between the control and library samples were screened by colony PCR with CKG-105/115, to amplify and Sanger sequence the *toxIN* locus. Mutations in *toxN* were identified by Sanger sequencing and re-introduced into fresh wild-type pBR322-*toxIN* by around-the-horn PCR (see above) to validate and further characterize.

### Phage spotting experiments

Phage spotting assays were conducted as described previously in (28). Briefly, bacterial strains of interest were mixed with LB + 0.5% agar (supplemented with 100 ng/ml aTc when necessary to induce *tifA* expression) and spread onto an LB + 1.2% agar + antibiotic plate. Phage stocks were serially diluted 10-fold in 1x FM buffer (20 mM Tris–HCl pH 7.4, 100 mM NaCl, 10 mM MgSO_4_) or LB and spotted on the bacterial lawn. Plates were then incubated overnight at 37°C before imaging. Images shown in figures are representative of at least three independent biological replicates.

### Co-immunoprecipitation assays to study ToxN-TifA interactions

Co-immunoprecipitation assays were performed as described in (28). Briefly, cultures of *E. coli* MG1655 containing pBR322-*toxIN*-His_6_ (wild-type or mutant) and pKVS45-*tifA_T4_*-FLAG (wild-type or mutant) were grown at 37°C in LB medium supplemented with 15 μg/ml L-tryptophan and 1 μg/ml thiamine to OD_600_= 0.4 and then infected with T4 at MOI = 5. Cells were treated with aTc at a final concentration of 100 ng/ml to induce production of TifA 30 min prior to T4 infection. After 15 min of T4 infection, 40 ml of cells from each culture were collected and pelleted by centrifugation (5 min at 4000 *g*, 4°C). After removing the supernatant, pellets were flash-frozen and stored at -80°C.

To perform anti-FLAG co-IP experiments, cell pellets were resuspended in 500 μl lysis buffer (25 mM Tris–HCl pH 8, 150 mM NaCl, 1 mM EDTA pH 8, 1% Triton X-100, cOmplete protease inhibitor cocktail (Roche), 1 μl/ml ReadyLyse (Novagen), 1 μl/ml DNase I (TakaraBio), 5% glycerol) and lysed by two freeze-thaw cycles followed by incubation on an end-to-end rotor for 1 h at 30°C. Lysates were clarified by centrifugation (10 min at 14000 *g*, 4°C), added to anti-FLAG-M2 magnetic beads (Sigma), and incubated with the beads for 1 h at 4°C while rotating on an end-to-end rotor. The beads were washed three times with wash buffer (25 mM Tris–HCl pH 8, 150 mM NaCl, 1 mM EDTA pH 8, cOmplete protease inhibitor cocktail (Roche), 5% glycerol), and the protein was eluted from the beads by adding 1x Laemmli buffer to the beads, vortexing, and boiling at 95°C. Samples were then analyzed by western blot as described previously (28). Blots shown are representative of at least two independent biological replicates.

### Cell growth assays in liquid culture

Plate reader growth assays were used to compare the growth rates of *E. coli* MG1655 cells transformed with pBR322-*toxIN*, pBR322-*toxI-toxN(K111N)*, pBR322-*toxI-toxN(K111R)*, pBR322-*toxI-toxN(Y115H)*, and pBR322-*toxI-toxN(L118S)*. Overnight cultures were back-diluted 1:100 in 4 ml LB and grown to mid-log phase at 37°C while rotating in a roller drum. Cultures were then back-diluted to OD_600_ = 0.001 in 1.5 ml LB, and growth was measured in 24-well plates at 15 min intervals with orbital shaking for 6 h at 37°C on a plate reader (Biotek). Data reported are the mean of two technical replicates each for four independent growth curve experiments. Growth rates of each strain were calculated using the Curveball package (30) with growth curves fit to the Richards (generalized logistic) model.

### Protein purification

*ToxN-TifA_RB69_, ToxIN, and ToxN-toxI-TifA_RB69_:* Complexes containing ToxN were purified using a modified version of the IMPACT kit protocol (NEB). 2–4 L of T7 Express (NEB) cells overexpressing ToxN with a C-terminal intein fused to chitin-binding domain (CBD) (pTXB1-*toxN*), along with *toxI* (pACYC-*toxI*), untagged TifA_RB69_ (pACYC-*tifA_RB69_*), FLAG-tagged TifA_RB69_ (pACYC-*tifA_RB69_*-FLAG), untagged TifA_RB69_ and *toxI* (pACYCDuet-1-*toxI-tifA_RB69_*), untagged TifA_RB69_(K55A R56A R59A) and *toxI* (pACYCDuet-1-*toxI-tifA_RB69_(K55A R56A R59A)*), or FLAG-tagged TifA_RB69_ and *toxI* (pACYCDuet-1-*toxI-tifA_RB69_*-FLAG) were grown in LB at 37°C to OD_600_ ∼ 0.8. Protein overproduction was then induced with the addition of 0.4 mM IPTG, and cultures were grown for an additional ∼18 h at 16°C. Cells were then harvested by centrifugation, resuspended in lysis/column buffer (20 mM Tris–HCl pH 8.5, 500 mM NaCl, 1 mM EDTA, 0.05% Tween-20, SIGMA*FAST* Protease Inhibitor Tablet (Sigma), 5% glycerol), and lysed using a Microfluidizer (three passes, 18 000 psi). The cell lysate was clarified by centrifugation at 15 000 rpm for 30 min and passed over chitin resin (NEB) pre-equilibrated with lysis/column buffer at 4°C. The resin was then washed with 25 column volumes of lysis/column buffer. To induce self-cleavage of the C-terminal intein-CBD tag, the resin was quickly washed with 3 column volumes of lysis/column buffer supplemented with 50 mM DTT (column cleavage buffer), capped, and incubated overnight at 4°C. The next day, 5 column volume of lysis/column buffer was added to each column to elute untagged ToxN and its binding partners. Fractions containing protein were pooled, concentrated through an Amicon 3K Centrifugal Filter Unit, and then passed over a Superose 6 Increase 10/300 GL column (GE Healthcare) equilibrated in column buffer (20 mM Tris–HCl pH 8.5, 50 mM NaCl, 1 mM EDTA, 5% glycerol). Fractions containing ToxN and/or TifA were identified by SDS-PAGE/Coomassie staining, concentrated, flash-frozen, and stored at −80°C. Fractions from the ToxN-TifA_RB69_-FLAG and ToxN-*toxI*-TifA_RB69_-FLAG purifications were analyzed by western blot as described previously (19,28), using mouse anti-FLAG antibodies (Sigma). The SuperSignal West Femto Maximum Sensitivity Substrate (ThermoFisher) was used to develop blots, and blots were imaged with the ChemiDoc MP Imaging System (Bio-Rad). The rough molecular weight of the ToxN-TifA-RNA peak on each column was estimated based on published standards (GE Healthcare). Each purification was performed and analyzed by FPLC at least three independent times.

*TifA-MBP-His_6_:* 1–4 L of T7 Express (NEB) cells overproducing TifA-MBP-His_6_ (pET-*tifA_RB69_*-MBP-His_6_) were grown in LB at 37°C to OD_600_ ∼0.8. Protein overproduction was then induced with the addition of 0.4 mM IPTG, and cultures were grown for an additional ∼18 h at 16°C. Cells were then harvested by centrifugation, resuspended in lysis buffer (50 mM Tris–HCl pH 7.5, 500 mM NaCl, 0.05% Tween-20, 1 mM TCEP, 0.18 mg/ml PMSF, SIGMA*FAST* Protease Inhibitor Tablet (Sigma), 5% glycerol) and lysed using a Microfluidizer (three passes, 18000 psi). The cell lysate was clarified by centrifugation at 15 000 rpm for 30 min and passed over Ni-NTA agarose resin (QIAGEN) pre-equilibrated with buffer A (50 mM Tris–HCl pH 7.5, 500 mM NaCl, 1 mM TCEP, 0.18 mg/ml PMSF, SIGMA*FAST* Protease Inhibitor Tablet (Sigma), 5% glycerol, 10 mM imidazole). The resin was then washed with 25 column volumes of buffer A. The protein was eluted from the resin with 5 column volumes of buffer B (50 mM Tris–HCl pH 7.5, 500 mM NaCl, 1 mM TCEP, 0.18 mg/ml PMSF, SIGMA*FAST* Protease Inhibitor Tablet (Sigma), 5% glycerol, 300 mM imidazole). Fractions containing protein were pooled and then concentrated and buffer-exchanged into buffer B without imidazole or protease inhibitor using an Amicon 10K Centrifugal Filter Unit. Purified protein was then passed over a Superose 6 Increase 10/300 GL column (GE Healthcare) equilibrated in column buffer (20 mM Tris–HCl pH 8.5, 50 mM NaCl, 1 mM EDTA, 5% glycerol). Fractions containing TifA-MBP-His_6_ were identified by SDS-PAGE/Coomassie staining, concentrated, flash-frozen, and stored at −80°C. TifA(P53A T54A)-MBP-His_6_ and TifA(K55A R56A R59A)- MBP-His_6_ were purified analogously. Each purification was performed and analyzed by FPLC at least two independent times.

### TifA-nucleic acid *in vitro* binding assay using fluorescence polarization

TifA binding activity to RNA, ssDNA, and dsDNA was tested using a fluorescence polarization-based method. *In vitro* synthesized TifA_RB69_ was purchased from AAPPTEC and solubilized in MilliQ H_2_O prior to use in *in vitro* binding experiments. 5′ 6-FAM (fluorescein)-labeled nucleic acid molecules derived from the region surrounding the ToxN cut site in the *E. coli* transcript *artJ* (RNA oligos CKG 658–660 and ssDNA oligos CKG-674–675) and from the 16S rRNA (RNA oligos CKG-676–678) were purchased from IDT and solubilized in 10 mM Tris–HCl, pH = 7.5, 0.1 mM EDTA. To construct dsDNA fragments derived from *artJ*, CKG-674 and CKG-675 were annealed to complementary unlabeled 30 and 45 nt oligos, respectively, purchased from IDT. Binding reactions were assembled in a final volume of 20 μl in 1× binding buffer (150 mM NaCl, 50 mM Tris–HCl, pH 7.5, 1 mM DTT, 5% glycerol, 0.1 mg/ml ultrapure BSA (Ambion)) with varying concentrations of TifA_RB69_ and 5 nM RNA or DNA. Reactions were incubated for 30 min at 37°C in a black flat bottom 384-well plate (Corning 3573). Fluorescence polarization was measured with a Tecan Infinite 200 Pro F Plex plate reader with 485 nm/20 nm bandwidth excitation filters and 530 nm/25 nm bandwidth emission filters fitted with polarizers. Data were fit in GraphPad Prism using the following equation: FP_obs_ = [Protein]*FP_max_ + *K*_D_*FP_min_ / [Protein] + *K*_D_. Data shown are the result of three independent biological replicates.

### RNA-seq following overexpression of ToxN-*toxI*-TifA_RB69_ and ToxN-*toxI*-TifA_RB69_(K55A R56A R59A)

Whole-cell lysate RNA was isolated from T7 Express cells overproducing ToxN-*toxI*-TifA_RB69_-FLAG (which behaved analogously to cells overexpressing ToxN-*toxI*-TifA_RB69_, see Figure 6A versus Supplementary Figure S7B) or ToxN-*toxI*-TifA_RB69_(K55A R56A R59A) following lysis with a Microfluidizer via subsequent rounds of extraction with acid-buffered phenol, acid-buffered phenol chloroform, and chloroform, followed by isopropanol precipitation. RNA was isolated from purified proteins following elution from chitin resin via subsequent rounds of extraction with acid-buffered phenol chloroform and chloroform, followed by isopropanol precipitation. Each purification was performed in biological duplicate. 200 ng of each RNA sample was then used to make RNA-seq libraries using the NEBNext® Ultra™ II RNA Library Prep Kit for Illumina® following the manufacturers’ protocol for use with purified mRNA or rRNA-depleted RNA. Paired-end sequencing of the libraries was performed on an Illumina NextSeq 5000 machine at the MIT BioMicroCenter. FASTQ files were then mapped to the *E. coli* BL21(DE3) genome (NZ_CP053602.1) and plasmid maps for pTXB1-*toxN* and pACYC Duet-1-*toxI-tifA_RB69_* as previously described (19,31).

For analysis of reads mapping to each gene, one count was added to the middle of each read. All reads mapping to a given genomic region were then summed and normalized by reads per million (RPM). This quantity was then used for downstream analyses. For analysis of fragment density across 16S rRNA loci, one count was added to every position in each read. Counts at each position were then divided by a sample size factor as described in (31).

## Results

### TifA is a small phage protein that inhibits ToxN and its homologs

Producing TifA_T4_ from an inducible promoter rescues cell growth when *E. coli* ToxN is produced from a separate inducible promoter, suggesting that TifA_T4_ is a *bona fide* antitoxin for ToxN (Figure 1B) (28). TifA homologs from the closely-related T4-like coliphages T2, T6 and RB69 are also capable of rescuing the growth defects resulting from *E. coli* ToxN, hereafter referred to as ToxN_Ec_ when necessary for clarity (Figure 1B) (28). Although TifA_T2_ and TifA_T6_ are closely related to TifA_T4_ (>96% identity), TifA_RB69_ is more distantly related (56% identity).

To determine whether inhibition extended beyond these proteins, we constructed a panel of six additional TifA homologs from *E. coli* and *Pectobacterium atrosepticum* phages spanning seven phylogenetic clusters, as well as six additional type III toxins—five ToxN homologs and one TenpN homolog—from different environmental isolates of γ-proteobacteria species for which phages contain *tifA* homologs (Supplementary Figure S1A-S1B, Supplementary Table S2). We cloned each *tifA* and *toxN* homolog into vectors that allow for separate and inducible expression and co-transformed these plasmids into *E. coli* MG1655. We then conducted a pairwise neutralization screen on plates that induced production of both ToxN and TifA (Figure 1C, Supplementary Figure S2A-F). We judged rescue of toxicity by comparing the growth of each strain producing a given toxin +/– TifA induction.

We found that the TifA homologs from T2, T4, T6 and RB69 displayed relatively broad inhibitory activity. In addition to neutralizing ToxN_Ec_, these TifA homologs could neutralize three other closely-related ToxNs (≥ 81% sequence similarity to ToxN_Ec_) from *P. atrosepticum*, *Klebsiella pneumoniae*, and *Shigella sonnei*, but not the more distantly related ToxN homologs from *Vibrio kanaloae* and *Serratia* sp. SRS-8-S-2018 (< 33% sequence similarity to ToxN_Ec_) or *E. coli* TenpN. The TifA homologs from other *E. coli* and *P. atrosepticum* phages did not neutralize any of the ToxN homologs or TenpN. To verify that a lack of rescue was not caused by low expression of TifA homologs, we used immunoblotting to confirm for two homologs that they were produced at high levels (Supplementary Figure S2G). We conclude that TifA homologs display specificity in their inhibition of ToxN proteins, suggesting that TifA is not a nonspecific inhibitor of toxin RNase activity and likely interacts with sequence or structural features of ToxN.

### AlphaFold prediction of the ToxN–TifA interface

**Figure 2.**
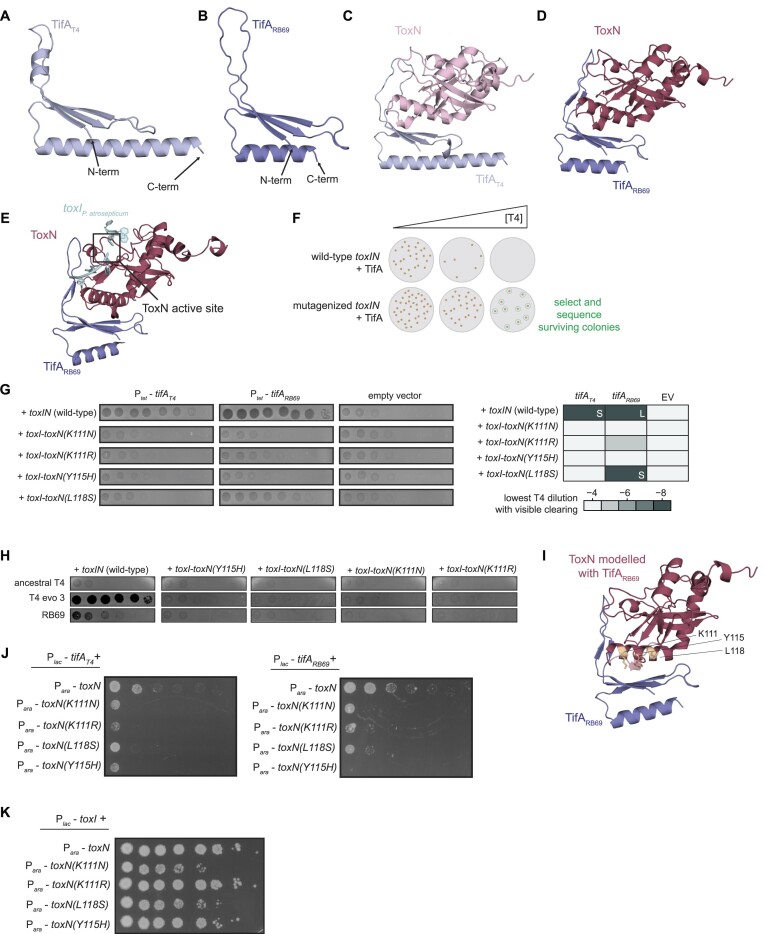
Prediction and validation of the ToxN-TifA interface. (**A, B**) AlphaFold model for TifA_T4_ (A) and TifA_RB69_ (B). (**C, D**) AlphaFold models for the ToxN-TifA_T4_ (C) and ToxN-TifA_RB69_ (D) complexes. (**E**) AlphaFold model for ToxN-TifA_RB69_ with *toxI_Pa_* and the predicted ToxN active site indicated. The position for *toxI_Pa_* relative to ToxN was visualized by overlaying the *E. coli* ToxN predicted structure and the ToxIN_Pa_ crystal structure (PDB: 2xdd) and is shown to indicate the ToxN active site. (**F**) Schematic of *tab* screen used to isolate TifA-resistant ToxN mutants. (**G**) (*Left*) Spotting assays for T4 on lawns of +*toxIN* (either wild-type or mutant *toxN*) cells with TifA_T4_, TifA_RB69_, or neither expressed from an anhydrous tetracycline (tet)-inducible promoter during phage infection. (*Right*) Heatmap quantifying spotting assays, with color indicating the lowest T4 dilution in which the cells displayed visible clearing (either plaques or lower cell density). L and S refer to large and small plaques, respectively, for samples on which T4 formed visible plaques. (**H**) Spotting assays for wild-type (ancestral) T4, evolved T4 with increased expression of *dmd*-*tifA* (T4 evo 3), and RB69 on lawns of +*toxIN* (with wild-type or mutant *toxN*) cells. (**I**) AlphaFold model for the ToxN-TifA_RB69_ complex, with residues K111, Y115 and L118 on ToxN highlighted. (**J**) Serial dilutions of *E. coli* cells ectopically expressing *toxN* (wild-type or mutant) and *tifA_T4_* or *tifA_RB69_*. (**K**) Serial dilutions of *E. coli* cells ectopically expressing *toxN* (wild-type or mutant) and *toxI*. Also see Supplementary Figures S3 and S4.

TifA_T4_ is sufficient, in the absence of any other phage protein, to neutralize ToxN, and prior work showed that ToxN co-precipitates with TifA_T4_ during T4 infection, suggesting that the two proteins interact (28). To investigate the molecular features of their interaction, we first used AlphaFold to predict the structures of TifA_T4_ and TifA_RB69_ (Figure 2A, B, Supplementary Figure S3A-S3B) (32). Each protein was predicted to adopt an L-shaped structure with an N-terminal three-stranded β-sheet packed against a C-terminal α-helix predicted with high confidence. Protruding from this base is an ∼20 amino acid-long loop region predicted with lower confidence.

We then used AlphaFold to model ToxN in complex with TifA_T4_ or TifA_RB69_ (Figure 2C, D). The AlphaFold models for ToxN in both complexes closely resembled the existing crystal structure (20) of *P. atrosepticum* ToxN (81% sequence similarity to ToxN_Ec_) solved in complex with its cognate *toxI* (RMSD of 1.87 and 0.91 Å, respectively) (Supplementary Figure S3C, D). Although AlphaFold yielded medium- to high-confidence predictions for ToxN and each of the two TifA homologs individually, the predictions for the interactions between the two proteins were less certain (Supplementary Figure S3F-I). Nevertheless, for both TifA_T4_ and TifA_RB69_, the highest-ranking models predicted that the interior of the L-shaped TifA interacted with ToxN, with several residues from the N-terminal β-sheet and the predicted 20 amino acid loop of TifA oriented toward the kinked α-helix and a loop region on the surface of ToxN (Figure 2C, D, Supplementary Figure S3E). We did not observe any large rearrangements of the predicted *E. coli* ToxN structure relative to the crystal structure of *P. atrosepticum* ToxN, indicating that TifA likely does not alter the conformation of ToxN (Supplementary Figure S3C, D). However, part of the region on ToxN predicted to be bound by TifA overlaps a region of ToxN involved in RNA substrate binding, and the tip of the 20 amino-acid loop in TifA appears to partially occlude the ToxN active site (Figure 2E), suggesting that TifA may directly interfere with ToxN-mediated cleavage of RNA targets.

### ToxN can escape TifA via mutations in the *toxI* binding pocket

To corroborate the AlphaFold predictions for the ToxN-TifA interface, we conducted a screen for mutants of ToxN that could escape TifA. Using error-prone PCR, we mutagenized *toxN* in the context of plasmid-borne *toxIN* under the control of its native promoter. We then transformed this library into *E. coli* MG1655 containing plasmid-borne TifA_T4_ or TifA_RB69_ under the control of a tetracycline-inducible promoter (P*_tet_*-*tifA*). We previously showed that high levels of TifA_T4_ or TifA_RB69_ are sufficient to prevent *toxIN*-mediated defense against T4 infection, leading to propagation of T4. Thus, we reasoned that mutations in *toxN* that disrupt the ToxN-TifA interface without abolishing ToxN activity would restore the ability of ToxN to block T4 infection.

Because ToxIN is an abortive infection (Abi) system, it is impossible to directly select for cells with functional ToxIN systems following phage infection. Instead, we used a selection strategy called the *tab* (T4 abortive) method, which allows for isolation of microcolonies with functional Abi systems following phage infection (Figures 2F, S4A) (16,33). We challenged our library of *toxIN* mutants, in the context of TifA_T4_ or TifA_RB69_ overproduction, with increasing concentrations of T4, mixed the cells and phage with soft agar, and allowed infection to proceed overnight. Because of limited phage diffusion through the agar, cells containing functional ToxIN systems could form microcolonies on plates containing low and intermediate concentrations of T4. We identified concentrations of T4 in which our *toxIN* mutant library, but not cells containing wild-type *toxIN*, formed microcolonies and Sanger sequenced approximately 40 surviving colonies for each TifA homolog, which resulted in 12 unique mutations. We re-cloned each mutation into pBR322-*toxIN* and transformed each plasmid into *E. coli* MG1655 containing plasmid-borne *P_tet_*-*tifA_T4_* or *P_tet_*-*tifA_RB69_*. We infected these strains with T4 to verify that each mutant *toxN* could indeed overcome TifA, using inhibition of T4 plaquing as a readout. Of the twelve original mutations, four were verified to confer defense against T4 infection in the presence of overproduced TifA (Figure 2G). There were three substitutions in ToxN that rendered cells partially or completely resistant to both TifA_T4_ and TifA_RB69_ (K111N, K111R, and Y115H) and one substitution that rendered cells resistant to only TifA_T4_ (L118S).

We then tested the ability of each *toxN* mutant to avoid phage-encoded TifA (as opposed to TifA produced from an inducible promoter as in Figure 2G). We transformed pBR322-*toxIN* plasmids containing each mutation into *E. coli* MG1655 and then, for each strain, compared the plaquing efficiency of several phages (Figure 2H). Both RB69 and an evolved T4 clone (T4 evo 3 (28)) containing an amplification of *tifA* could form plaques on wild-type +*toxIN* cells, indicating that TifA was inhibiting ToxN. However, like wild-type T4, both RB69 and T4 evo 3 were restricted in the presence of the four ToxN mutants. Thus, the substitutions K111N, K111R, Y115H, and L118S in ToxN allow it to evade inhibition by TifA during phage infection.

The three residues we identified in the screen (K111, Y115, and L118) are localized on the surface of the kinked α-helix in ToxN within the ToxN-TifA interface predicted by AlphaFold (Figure 2I), suggesting that these residues may help mediate the interaction between ToxN and TifA. To further test this hypothesis, we overexpressed each ToxN mutant in uninfected cells ectopically producing TifA_T4_ or TifA_RB69._ All four substitutions prevented TifA rescue of ToxN-mediated toxicity, supporting the notion that residues K111, Y115, and L118 are important for the antitoxin activity of TifA (Figure 2J).

Y115 and L118 were previously identified as residues mediating ToxN-*toxI* interactions in a closely-related ToxIN system from *P. atrosepticum*, and these residues map to the interface formed between *toxI* and ToxN in the crystal structure of *P. atrosepticum* ToxIN (20). To test whether the mutations found to disrupt ToxN-TifA interaction also weaken the ToxN-*toxI* interaction, we co-produced each ToxN mutant with *toxI* under separate, inducible promoters and monitored cell growth on medium that promotes expression of both *toxN* and *toxI* (Figure 2K). Although ToxN(K111R) and ToxN(Y115H) were as robustly inhibited by *toxI* as wild-type ToxN, ToxN(K111N) and ToxN(L118S) displayed significantly less sensitivity to *toxI*, resulting in a lower plating efficiency and smaller colonies. Thus, although substitutions in the *toxI* binding pocket allow ToxN to overcome TifA, they may also weaken the ability of *toxI* to sequester ToxN in uninfected cells. Despite this change in sensitivity to *toxI*, we did not observe obvious growth defects from *toxIN* constructs containing mutations in those positions when *toxIN* was expressed as an operon (Supplementary Figure S4B). However, *toxI* transcript levels greatly exceed those of *toxN* in the native locus (11) and thus may be sufficient to sequester mutant ToxN proteins during normal cell growth.

### Naturally-occurring substitutions in TifA enhance inhibition of ToxN

**Figure 3.**
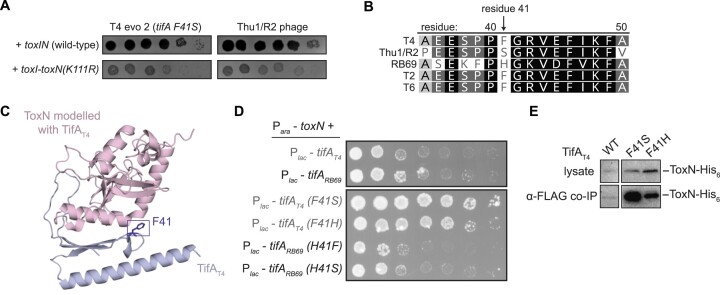
Mutations in TifA that strengthen its interaction with ToxN. (**A**) Spotting assays for evolved T4 phage with increased expression of *dmd-tifA(F41S)* (T4 evo 2) and Thu1/R2 phage on lawns of +*toxIN* and +*toxI-toxN(K111R)* cells. (**B**) Al**i**gnment of residues 35–50 of TifA homologs from several T4-like phages, with residue 41 indicated. Degree of sequence similarity is highlighted in gray/black. (**C**) Alp**haF**old model for the ToxN-TifA_T4_ complex, with residue F41 on TifA highlighted. (**D**) Serial dilutions of *E. coli* cells ectopically expressing *toxN* and wild-type or mutant *tifA_T4_* or *tifA_RB69_*. (**E**) Western blot of ToxN-His_6_ in cell lysate and following co-immunoprecipitation with TifA_T4_-FLAG (wild-type or mutant) during T4 infection.

At late rounds in our original evolution of T4 to overcome *toxIN*, we had noticed the emergence of a mutation producing the substitution F41S in TifA in one of our evolved populations (evo 2) that had an amplification of the *tifA-dmd* locus. These evolved phages were slightly better at infecting +*toxI-toxN(K111R)* cells than other lines of evolved phages (Figures 2H, 3A) suggesting that the F41S substitution strengthens the interaction between ToxN and TifA and restores neutralization of ToxN(K111R). Notably, a serine occurs naturally at position 41 in the TifA homolog from the T4-like coliphage Thu1/R2, which has a single copy of *tifA* but can replicate on both +*toxIN* and +*toxIN(K111R)* cells (Figure 3A-B). For TifA_RB69_, which is a more robust inhibitor of ToxN (Figures 1B, 3D), residue 41 is a histidine (Figure 3B). In both ToxN-TifA AlphaFold structures, residue 41 is on a loop in the predicted ToxN-TifA binding interface (Figure 3C). Thus, we hypothesized that substituting F41 with either serine or histidine improves the inhibition of ToxN by TifA_T4_.

To test this hypothesis, we examined whether TifA_T4_ (F41S or F41H) and TifA_RB69_ (H41S or H41F) could neutralize the toxicity of ToxN in uninfected cells (Figure 3D). Both substitutions in TifA_T4_ substantially increased its ability to inhibit ToxN relative to wild-type TifA_T4_. Conversely, for TifA_RB69_, H41F and H41S both appeared to modestly decrease inhibition of ToxN, as manifested by smaller colonies at the lowest plating dilution with visible colonies. We also performed an anti-FLAG IP on cells producing FLAG-tagged TifA_T4_ and ToxN-His_6_ during T4 infection (Figure 3E). Immunoblotting for ToxN-His_6_ revealed much stronger signal for the samples in which TifA harbored either the F41S or F41H substitution compared to wild-type TifA. Taken together, these observations indicate that position 41 of TifA, which is predicted to be positioned at the ToxN-TifA interface and varies in TifA homolog sequences, mediates inhibition of ToxN.

### ToxN and TifA form an RNA–protein complex

**Figure 4.**
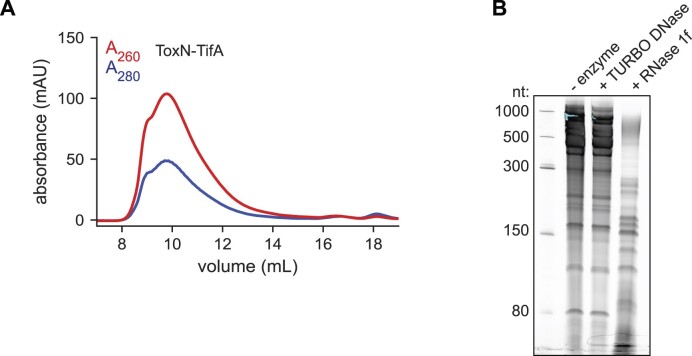
ToxN and TifA form an RNA-protein comple**x**.(**A**) Size-exclusion chromatogram of purified ToxN-TifA_RB69_ run on a Superose 6 Increase 10/300 GL column. (**B**) Denaturing 6% urea-PAGE gel resolving nucleic acid that copurifies with ToxN-TifA_RB69_ on chitin resin; nucleic acid was treated with Turbo DNase, RNase 1f, or neither before running on the gel. Also see Supplementary Figure S5.

Given that TifA is sufficient to neutralize ToxN and that the proteins interact in co-IP experiments, we sought to overexpress and purify a ToxN-TifA complex to further characterize the interactions between the two proteins. We focused on the ToxN-TifA_RB69_ complex as TifA_RB69_ is a stronger inhibitor of ToxN than TifA_T4_ (Figure 2G, H). We tagged ToxN with a chitin-binding domain and, after confirming that this fusion protein was functional and rescued by TifA_RB69_ (Supplementary Figure S5A), co-expressed it with untagged TifA_RB69_ in *E. coli* BL21. After column purification on chitin agarose resin, we used size exclusion chromatography with a Superose 6 column to isolate the complex. We assessed nucleic acid and protein levels in our eluent by monitoring absorbance at 260 and 280 nm, respectively (Figure 4A). To our surprise, a major peak appeared in the void volume of the column. By repeating the ToxN-TifA purification in a strain producing FLAG-tagged TifA, which is functional (Supplementary Figure S5A), we confirmed the presence of TifA in the high molecular weight peak using western blotting (Supplementary Figure S5B). The major peak's ratio of absorbance at 260 nm to absorbance at 280 nm (A_260_/A_280_) was close to 2, suggesting that it contained both protein and nucleic acid. We extracted nucleic acid from the eluent of the chitin agarose purification, treated it with DNase or RNase, ran it on a denaturing urea-PAGE gel, and stained the gel with SYBR Gold to visualize both DNA and RNA (Figure 4B). The co-localizing nucleic acid was mostly high molecular weight material (>300 nucleotides) and was resistant to DNase treatment and sensitive to RNase treatment, suggesting that it was RNA (Figure 4B).

### TifA is an RNA-binding protein

**Figure 5.**
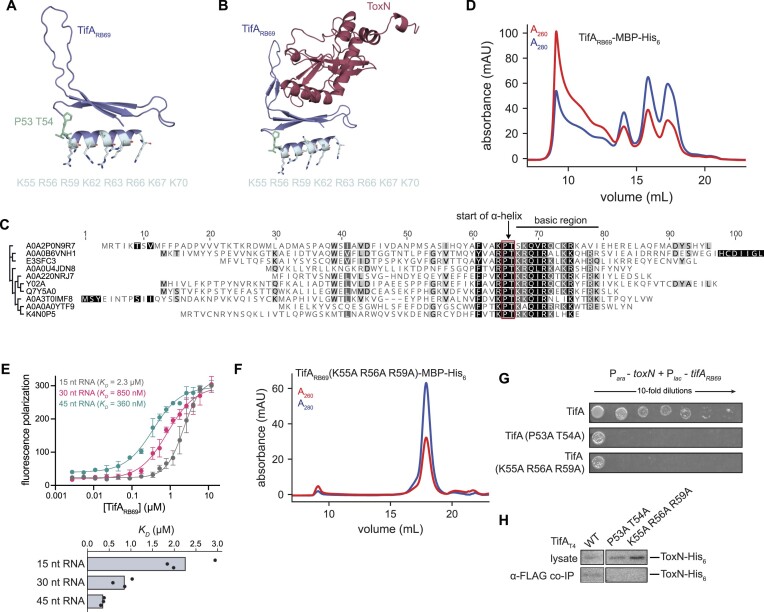
TifA is an RNA-binding protein. (**A**) AlphaFold model for TifA_RB69_ with residues capping the predicted C-terminal α-helix (P53 T54) and basic residues on the surface of the predicted α-helix indicated. (**B**) AlphaFold model for the ToxN-TifA_RB69_ complex with residues capping the predicted C-terminal α-helix (P53 T54) and basic residues on the surface of the predicted α-helix indicated. (**C**) Sequence alignment for 10 TifA homologs from different phylogenetic clusters indicating the highly-conserved PT motif, immediately followed by a cluster of basic residues. UniProtKB accession numbers for the TifA homologs in the alignment are, from top to bottom, as follows: A0A2P0N9R7; A0A0B6VNH1; E3SFC3; A0A0U4JDN8; A0A220NRJ7; Y02A; Q7Y5A0; A0A3T0IMF8; A0A0A0YTF9; K4N0P5. (**D**) Size-exclusion chromatogram of purified TifA_RB69_-MBP-His_6_ run on a Superose 6 Increase 10/300 GL column. (**E**) Fluorescence polarization assay of increasing concentrations of synthesized TifA_RB69_ incubated with several fluorescently-labeled RNA species (lengths indicated). RNA sequences were derived from the region of the *E. coli* transcript *artJ* that is cleaved by ToxN; see Supplementary Table S5 for sequences. (**F**) Size-exclusion chromatogram of purified TifA_RB69_ (K55A R56A R59A)-MBP-His_6_ run on a Superose 6 Increase 10/300 GL column. (**G**) Serial dilutions of *E. coli* cells ectopically expressing *toxN* and wild-type or RNA-binding deficient *tifA_RB69_*. (**H**) Western blot of ToxN-His_6_ in cell lysate and following co-immunoprecipitation with TifA_T4_-FLAG (wild-type or RNA-binding deficient) in T4-infected cells producing TifA_T4_ and ToxN. Note that the TifA_T4_ wild-type control is same as in Figure 3E because both experiments were run on the same blot. Also see Supplementary Figure S6.

We suspected that the TifA in ToxN–TifA complexes may directly bind RNA as the AlphaFold models of both TifA_T4_ and TifA_RB69_ predicted a C-terminal α-helix with a high density of solvent-exposed, basic residues, reminiscent of a nucleic acid binding motif (Figure 5A, B). The basic C-terminal region of TifA, as well as the Pro-Thr amino acids capping the α-helix in both TifA_T4_ and TifA_RB69_, are conserved in TifA homologs, further suggesting that this basic α-helix plays a crucial role in TifA function (Figures 5C, S6A). In contrast, the sequence of the loop region containing residue 41 in TifA_T4_ and TifA_RB69_ was conserved within closely-related sequences but not in wider taxonomic alignments (Supplementary Figure S6A). The sequence of this region, as opposed to the basic α-helix, may have evolved to interact with particular groups of ToxN homologs. To characterize TifA’s potential RNA binding activity, we overexpressed and purified TifA_RB69_-MBP-His_6_ on nickel resin and then ran purified protein on a size-exclusion column (Figure 5D). We confirmed that TifA_RB69_-MBP-His_6_ was a functional inhibitor of ToxN using cell growth assays (Supplementary Figure S6C). TifA-MBP-His_6_ eluted in several peaks, including one peak in the void volume of the column that was accompanied by an A_260_/A_280_ ratio of ∼2.0, as with the ToxN-TifA complex. Indeed, TifA_RB69_-MBP-His_6_ co-purified with RNA of a range of molecular weights (Supplementary Figure S6B), suggesting that TifA interacts with RNA in the absence of ToxN.

To confirm this finding, we tested the ability of TifA protein to bind nucleic acid *in vitro*. We incubated increasing concentrations of directly synthesized, untagged TifA_RB69_ with fluorescently-labeled RNAs of different lengths (15, 30 and 45 nucleotides) derived from the *E. coli* gene *artJ* and measured binding via fluorescence polarization (Figure 5E). TifA_RB69_ interacted with all three fragments, with measured *K*_D_ values of 2.3 μM, 850 nM and 360 nM for the 15, 30 and 45 nucleotide RNAs, respectively. TifA_RB69_ also interacted with several RNA fragments derived from the 16S rRNA (Supplementary Figure S6D). We also found that TifA bound short ssDNA and dsDNA fragments, with a similar affinity (Supplementary Figure S6D), but because the nucleic acid that co-precipitated with TifA was RNase sensitive and not DNase sensitive (Supplementary Figure S6B), we conclude that TifA primarily interacts with RNA *in vivo*.

To determine whether TifA’s predicted C-terminal, basic α-helix facilitates RNA binding, we overexpressed and purified a variant of TifA_RB69_-MBP-His_6_ in which we substituted the Pro-Thr residues capping the α-helix (P53A T54A) and a variant in which we substituted three basic residues in the α-helical region (K55A R56A R59A) (Figure 5F, Supplementary Figure S6E). Unlike wild-type TifA_RB69_, these variants each ran as a single, well-defined peak on a size-exclusion column. The major peak for both variants had an A_260_/A_280_ ratio close to 0.5, suggesting that they no longer co-purify with RNA.

Finally, we asked whether the RNA-binding ability of TifA was necessary for neutralization of ToxN. Unlike wild-type TifA_RB69_, neither TifA_RB69_(P53A T54A) nor TifA_RB69_(K55A R56A R59A) could rescue the cell growth defects of overproducing ToxN from a separate, inducible promoter (Figure 5G). Further, neither TifA_T4_(P53A T54A)-FLAG nor TifA_T4_(K55A R56A R59A)-FLAG co-immunoprecipitated with ToxN-His_6_ during T4 infection (Figure 5H), suggesting that these variants of TifA are no longer functional inhibitors and that RNA binding by TifA is necessary for ToxN-TifA interactions. Taken all together, our results indicate that TifA uses its C-terminal, basic α-helix to bind RNA and neutralize ToxN.

### ToxN co-purifies with 16S rRNA in the presence of TifA

**Figure 6.**
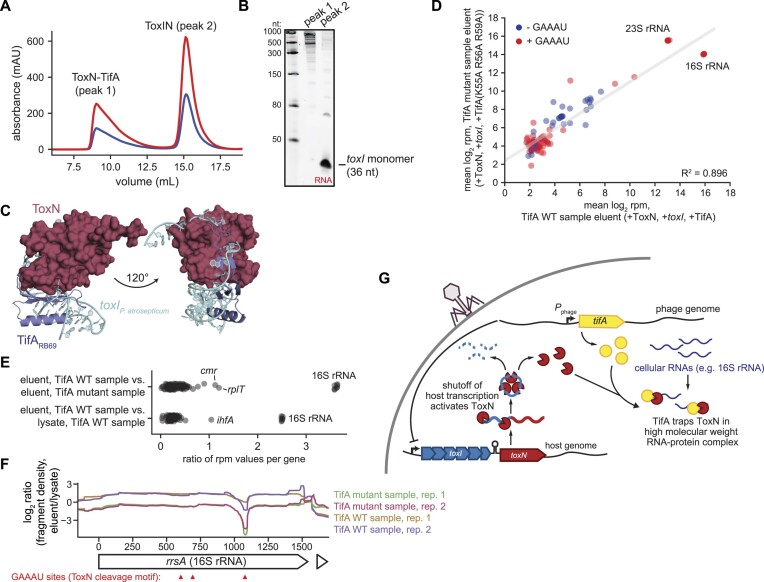
ToxN co-purifies with the 16S rRNA in the presence of TifA. (**A**) Size-exclusion chromatogram of ToxN co-purified with *toxI* and TifA run a Superose 6 Increase 10/300 GL column. (**B**) 6% urea–PAGE gel analyzing the RNA content of the two major peaks in (A). (**C**) AlphaFold model for the ToxN-TifA_RB69_ complex, with *P. atrosepticum toxI* (PDB: 2xdd) modelled into the complex. (**D**) Scatter plot comparing the mean summed RNA-seq counts (log_2_ RPM) for individual genes in the TifA WT sample eluent (+ToxN, +*toxI*, +TifA_RB69_) and the TifA mutant sample eluent (+ToxN, +*toxI*, +TifA_RB69_ (K55A R56A R59A)) (two biological replicates each). Genes with RPM values greater than or equal to 3 in all sequenced libraries (lysates and eluents) are shown. The gray line indicates the linear regression fit for the data. (**E**) Scatter plots showing the ratio of mean RPM values for individual transcripts in the samples indicated. (**F**) Plot of log_2_ (ratio, fragment density in eluent versus lysate) across the 16S rRNA locus *rrsA* for both biological replicates of the TifA WT and TifA mutant samples. Sites containing the ToxN cleavage motif (GAAAU) are indicated with red arrows. (**G**) Proposed model for TifA inhibition of ToxN. Following phage infection, shutoff of host transcription, including of the *toxIN* locus, leads to degradation of *toxI* and release of ToxN. TifA then interacts with activated ToxN to prevent degradation of cellular transcripts, likely by recruiting and/or sequestering ToxN to a subset of cellular RNAs, in particular the 16S rRNA, such that ToxN can no longer cleave phage transcripts. Also see Supplementary Figure S7.

During normal cell growth, ToxN is sequestered via interactions with its cognate RNA antitoxin, *toxI*. Based on our result that ToxN-TifA_RB69_ formed a ribonucleoprotein complex, we wondered whether TifA might form a ternary complex with ToxN and processed, 36-nucleotide *toxI* during T4 infection to stabilize the heterohexameric ToxIN complex. To test this idea, we overexpressed chitin binding domain (CBD)-tagged ToxN with untagged TifA_RB69_ and *toxI* and then purified ToxN and its binding partners by running the cell lysate through chitin agarose resin. We analyzed the purified proteins by size exclusion chromatography, again assessing nucleic acid and protein levels in our eluent (Figure 6A). We observed two major peaks – one in the void volume (peak 1) and one centered at ∼15.5 ml (peak 2)—both of which had an A_260_/A_280_ ratio close to 2. We analyzed the contents of each peak on an RNA gel. Peak 1, which resembled the copurification of ToxN and TifA alone, contained many high-molecular weight RNAs. In peak 2, which ran in the same location as purified ToxIN complex (Supplementary Figure S7A), the predominant RNA species was the same size as processed *toxI* (36 nucleotides) (Figure 6B). To test if TifA was associated with one of these peaks, we repeated the CBD-tagged ToxN purification from cells expressing *toxI* and FLAG-tagged TifA_RB69_ and then monitored the location of TifA_RB69_-FLAG via western blotting (Supplementary Figure S7B). TifA_RB69_-FLAG signal co-localized with the first, but not the second peak, indicating that TifA does not interact with heterohexameric ToxIN complexes. Taken together, these results indicate that *toxI* monomer and TifA interactions with ToxN are likely mutually exclusive, consistent with the AlphaFold prediction that TifA and *toxI* contact overlapping regions of ToxN (Figures 2D, 6C). Thus, TifA likely associates with ToxN after ToxN has dissociated from processed *toxI* but leads to formation of an RNA-protein complex that renders ToxN inert.

Next, we used RNA sequencing (RNA-seq) to identify the RNA species associated with CBD-tagged ToxN purified from cells expressing tagged ToxN, *toxI*, and TifA. We sought RNAs that were enriched following pull-down of ToxN co-expressed with *toxI* and TifA_RB69_ (hereafter called the TifA WT sample) relative to the cell lysate from that purification and relative to a purification of ToxN co-expressed with *toxI* and TifA_RB69_(K55A R56A R59A) (hereafter called the TifA mutant sample) that includes the TifA variant unable to bind RNA. We performed and sequenced two biological replicates of each pulldown, and sequencing reads were highly reproducible between biological replicates (Supplementary Figure S7C).

There was no evident enrichment of ToxN substrates in the TifA WT sample, indicating that ToxN cleavage of target transcripts does not guide the specificity of the ToxN-TifA-RNA complex (Figure 6D). In fact, the only RNA highly enriched in the TifA WT complex relative to either the TifA WT sample lysate or the TifA mutant sample eluent was the 16S rRNA (Figure 6D, E, Supplementary Figure S7D, E), which is transcribed from seven loci (*rrsA-G*) in the *E. coli* genome and comprises the RNA portion of the small ribosomal subunit. Because the sequences for the 16S rRNA loci are highly similar (>99% sequence similarity in *E. coli* BL21), RNA-seq reads for 16S rRNA were randomly distributed among the seven loci during our mapping process. Thus, we were unable to determine if one particular 16S rRNA locus was more highly enriched than others in our pulldown.

The fragment density counts for both the TifA WT sample and the TifA mutant sample (normalized to counts from their respective lysate samples) were roughly uniform across the 16S rRNA loci (example of *rrsA* shown in Figure 6F). However, in both samples, we noticed a depletion in fragment density near an instance of the ToxN cleavage motif (GAAAU) toward the 3′ end of the 16S rRNA, suggesting that ToxN might cleave co-purifying 16S rRNA. Notably, this cleavage valley was deeper in the TifA mutant samples compared to the TifA WT samples, providing additional evidence that TifA is a direct inhibitor of ToxN. To test whether the catalytic activity of ToxN is necessary for its interaction with TifA, we assessed TifA_T4_ binding to a nuclease-inactive version of ToxN (ToxN(K55A)) during T4 infection. ToxN(K55A) co-immunoprecipitated with TifA_T4_ with the same efficiency as wild-type ToxN (Supplementary Figure S7F), demonstrating that RNA cleavage by ToxN is not necessary for its sequestration by TifA. In sum, these findings suggest that TifA stabilizes an interaction of ToxN with 16S rRNA, with TifA binding RNA and recruiting ToxN or with TifA tethering ToxN to RNA molecules during ToxN’s target search process (Figure 6G). These stabilizing interactions likely trap ToxN in an inactive state, thereby preventing it from acting as an effective, multiple turnover RNase.

## Discussion

Bacteria and phages are locked in an ongoing coevolutionary arms race, with each rapidly evolving mechanisms to subvert or overcome the other (2–4,34). Toxin-antitoxin (TA) systems are widespread antiphage defense elements in bacteria in which activation of the toxin during infection prevents phage replication and propagation through the rest of the bacterial population (6,9). Phages have been shown to overcome TA systems by several means, including by producing *bona fide* antitoxins from their own genomes and by preventing toxin activation (13,14,25,28,35). We recently discovered a small protein in T4 and T4-like phages, called TifA, that interferes with ToxN-mediated antiphage defense (28). The cognate antitoxin for ToxN is a short RNA molecule, suggesting that TifA must inhibit ToxN differently than *toxI*.

Here, we found that TifA is a small RNA-binding protein that forms an RNA-protein complex with ToxN when they are co-produced. Our observation that TifA proteins display specificity for certain ToxN homologs suggests that TifA does not function by simply coating RNA to prevent any RNase degradation; instead, its inhibition is likely mediated by specific protein-protein interactions with ToxN. Our AlphaFold predictions for the ToxN-TifA structure, and subsequent characterization of TifA-resistant ToxN mutants and naturally-occurring TifA variants, corroborate this hypothesis. We identified several residues localized in the predicted ToxN-TifA interface that strengthen or weaken TifA inhibition of ToxN, providing evidence for specific molecular interactions between ToxN and TifA. However, AlphaFold predicted several orientations for ToxN and TifA relative to one another, making the identification of precise protein-protein interaction points difficult. One possibility for why AlphaFold did not predict a single, high-confidence structure is that the complex requires the presence of RNA, which was not included in the modeling. Interestingly, two of the mutations that we found in ToxN that allow it to overcome TifA also weaken its interaction with its cognate antitoxin, *toxI*. Thus, *toxIN* may face a tradeoff in acquiring mutations that prevent inhibition by TifA, as doing so might also risk liberation of ToxN from *toxI* in the absence of phage infection.

Based on our results, we propose the following model for TifA inhibition of ToxN, using T4 infection as an example (Figure 6G). Following phage infection, the shutoff of host transcription, including the *toxIN* locus, leads to degradation of *toxI* precursors and release of ToxN from processed, monomeric *toxI* (19). *tifA* is an early gene in T4, so it is rapidly expressed once the T4 genome is injected into the infected cell. TifA then interacts with activated ToxN to prevent degradation of cellular transcripts, likely by trapping ToxN on 16S rRNA, and possibly other abundant cellular RNAs. In a previous study, we used RNA-seq to identify ToxN targets in uninfected *E. coli* cells (19). In this context, the 16S rRNA was not cleaved by ToxN, suggesting that TifA may determine the binding specificity of the ToxN-TifA complex. In sum, we propose that TifA effectively prevents ToxN from operating as an efficient, multiple-turnover RNase.

### TifA as a phage-encoded antitoxin

In our model for TifA activity, it does not act like a type II protein antitoxin that binds to and neutralizes a cognate toxin in the absence of other cofactors (8). Instead, inhibition involves both a protein (TifA) and RNA. Interactions between toxins, their substrates, and inhibitory factors have been observed for other toxin–antitoxin and antiviral defense systems. For example, in the plasmid maintenance system CcdAB, the toxin CcdB binds to and inhibits DNA gyrase. Its cognate antitoxin, CcdA, can bind to the CcdB-gyrase complex to release gyrase-bound CcdB (36). Similarly, the anti-CRISPR protein AcrIIC1 from a *Neisseria meningitidis* mobile genetic element inhibits DNA-bound *N. meningitidis* Cas9 by preventing the conformational changes in the Cas9 active site required for DNA cleavage (37,38). Strikingly, it was recently discovered that the interaction between APOBEC3, a host-encoded antiviral protein, and HIV-1 Vif, an APOBEC3 antagonist, requires RNA as a ‘molecular glue’ to correctly position residues in Vif that inhibit APOBEC3. Analogous to our observation that residues in ToxN that mediate its interactions with TifA are also necessary for complete inhibition by *toxI*, the APOBEC3–Vif–RNA interface includes residues in APOBEC3 that are required for its restriction of viral replication in the absence of Vif (39). In the case of ToxN-TifA, it remains to be determined exactly how ToxN RNase activity is inhibited in the presence of TifA.

### Concluding remarks

The diversity of systems underlying antiphage defense and phage counter-defense is readily apparent; however, the molecular underpinnings of most of these systems remain poorly characterized. Having previously discovered TifA, an anti-*toxIN* element in T4-like phages, we investigated the mechanism underlying inhibition of an antiphage TA system by a phage-encoded antitoxin. Our results suggest that the RNA-binding protein TifA inhibits ToxN by sequestering it onto abundant RNAs in the cell, most notably the 16S rRNA, thereby rendering it inert. Thus, our results reveal a novel mechanism underlying phage inhibition of an RNase toxin and emphasize the ongoing arms race between T4-like phages and *toxIN* systems.

## Supplementary Material

gkad1207_Supplemental_File

## Data Availability

RNA sequencing data generated in this study is available in the Gene Expression Omnibus at https://www.ncbi.nlm.nih.gov/geo/ under accession code GSE234211. Funding for open access charge: HHMI funds.
