# Supplementary material for: A phage-encoded RNA-binding protein inhibits the antiviral activity of a toxin–antitoxin system
Source: Nucleic Acids Res. 2023 Dec 20;52(3):1298–312. doi: 10.1093/nar/gkad1207 (PMC10853763; doi:10.1093/nar/gkad1207)
Supplement: gkad1207_Supplemental_File [file gkad1207_supplemental_file.pdf]

Supplemental Files for:

A phage-encoded RNA-binding protein inhibits the antiviral activity of a toxin-antitoxin system

Chantal K. Guegler, Gabriella I.C. Teodoro, Sriram Srikant, Keerthana Chetlapalli, Christopher R. Doering, Dia A. Ghose, Michael T. Laub

Supplemental Figure and Table Legends

Figure S1. TifA and ToxN homologs are present in T4-like phage and bacterial genomes, respectively

(A) Protein homology tree based on primary sequence alignments of 57 TifA homologs, with the TifA homologs used for the screen in Figure 1C indicated. Note that $TifA_{T2}$ and $TifA_{T6}$ differ by only a few amino acids, and therefore only $TifA_{T6}$ was included in the tree.

(B) Multiple sequence alignment of ToxN homologs used for the screen in Figure 1C. Degree of sequence similarity is highlighted in gray/black.

Figure S2. Rescue of ToxN homolog overexpression by co-expression of various TifA homologs

(A-F) Cell spotting assays of *E. coli* MG1655 transformed with pBAD33-*toxN* and pEXT20-*tifA* used to assemble Figure 1C. To induce ToxN and TifA, cells were serially-diluted 10-fold and spotted on plates containing both IPTG and arabinose. To induce only ToxN, cells were spotted on plates containing only arabinose. A toxin was counted as rescued by a TifA homolog if, across multiple replicates, its colony-forming efficiency and/or colony size was increased on +arabinose, +IPTG (ToxN and TifA induced) plates relative to +arabinose (ToxN induced) plates. Bacterial species from which ToxN and TenpN homologs were cloned: *Escherichia coli* (Ec); *Pectobacterium atrosepticum* (Pa); *Klebsiella pneumoniae* (Kp); *Shigella sonnei* (Ss); *Vibrio kanaloae* (Vk); *Serratia* sp. SRS-8-S-2018 (Ser).

(G) Western blot of two FLAG-tagged TifA homologs ($TifA_{vB\_PatP\_CB4}$ and $TifA_{vB\_EcoM\_005}$) following production from an IPTG-inducible promoter for ~6.5 hours in liquid culture. RpoA is included as a loading control.

Figure S3. AlphaFold predictions of the TifA and ToxN-TifA structures

(A) Per-residue confidence of five AlphaFold predictions for the structure of $TifA_{T4}$. The top-ranked prediction was used for downstream analyses.

(B) Per-residue confidence of five AlphaFold predictions for the structure of $TifA_{RB69}$. The top-ranked prediction was used for downstream analyses.

(C) Overlay of the crystal structure of *P. atrosepticum* ToxN ($ToxN_{Pa}$) and the predicted structure of *E. coli* ToxN ($ToxN_{Ec}$) in complex with $TifA_{T4}$. The positions of ToxN residues K111, Y115, and L118 in the model of $ToxN_{Ec}$ are shown in magenta.

(D) Overlay of the crystal structure of *P. atrosepticum* ToxN ($ToxN_{Pa}$) and the predicted structure of *E. coli* ToxN ($ToxN_{Ec}$) in complex with $TifA_{RB69}$.

(E) Space-filling AlphaFold model for the ToxN-$TifA_{RB69}$ complex, with TifA residues predicted to interact with ToxN indicated.

(F) Per-residue confidence of five AlphaFold predictions for the structure of ToxN in complex with $TifA_{T4}$. The top-ranked prediction was used for downstream analyses.

(G) Per-residue confidence of five AlphaFold predictions for the structure of ToxN in complex with $TifA_{RB69}$. The top-ranked prediction was used for downstream analyses.

(H) Heat map showing predicted aligned error (PAE) for five AlphaFold-predicted models for the ToxN-$TifA_{T4}$ complex; a low PAE value indicates that AlphaFold predicts well-defined relative positions and orientations for the two domains or proteins. The highest-ranking model was used for analysis in the manuscript.

(I) Heat map showing predicted aligned error (PAE) for five AlphaFold-predicted models for the ToxN-$TifA_{RB69}$ complex. The highest-ranking model was used for analysis in the manuscript.

Figure S4. Use of a *tab* screen to isolate TifA-resistant ToxN mutants

(A) Example selection plates for +*toxIN*, +$TifA_{RB69}$ cells during *tab* selection. Serial 10-fold dilutions of T4 were mixed with cells expressing TifA from an inducible promoter and a library of *toxIN* variants (or wild-type *toxIN* as a control). Microcolonies in the *toxIN* library that arose at dilutions of T4 in which the control cells (+*toxIN* wild-type, top) no longer grew (both dilutions shown) were isolated and Sanger sequenced.

(B) Growth curves for MG1655 cells harboring pBR322-*toxIN* (wild type or mutant), with calculated doubling times indicated in brackets.

Figure S5. ToxN forms a high molecular weight RNA-protein complex when it is co-purified with TifA

(A) Serial dilutions of *E. coli* cells expressing *toxN* (wild-type or chitin-binding domain-tagged) and *tifA*$_{RB69}$ (wild-type or FLAG-tagged). Plasmids harboring *toxN* and *tifA* under arabinose- and IPTG-inducible promoters, respectively, were transformed into *E. coli* MG1655, and protein production was induced with the addition of arabinose and IPTG.

(B) (*top*) Size-exclusion chromatogram of purified ToxN-TifA$_{RB69}$-FLAG run on a Superose 6 Increase 10/300 GL column with collected fractions indicated. (*bottom*) Anti-FLAG Western blot probing for TifA$_{RB69}$-FLAG in various FPLC fractions, indicating that TifA$_{RB69}$ localizes in the high molecular weight peak.

Figure S6. TifA has a conserved nucleic acid-binding domain

(A) Alignment of 57 TifA homologs, with the position of F41 in TifA$_{T4}$, the conserved PT motif, and the cluster of basic residues indicated. Degree of sequence similarity is highlighted in gray/black.

(B) Denaturing 6% urea-PAGE gel resolving nucleic acid that copurifies with TifA$_{RB69}$-MBP-His$_6$ in Ni-NTA resin purification; nucleic acid was treated with Turbo DNase, RNase 1f, or neither before running on the gel.

(C) Serial dilutions of *E. coli* cells expressing *tifA*$_{RB69}$-MBP-His$_6$ with and without co-expression of *toxN*. Plasmids harboring *toxN* and *tifA* under arabinose- and IPTG-inducible promoters, respectively, were transformed into *E. coli* MG1655, and protein production was induced with the addition of arabinose and IPTG.

(D) Results of fluorescence polarization assays measuring the binding affinity of TifA$_{RB69}$ incubated to several fluorescently labeled RNA, ssDNA, and dsDNA species (lengths and sources of sequences indicated). See Table S5 for sequences of nucleic acid substrates tested.

(E) Size-exclusion chromatogram of purified $TifA_{RB69}$ (P53A T54A)-MBP-His_6 run on a Superose 6 Increase 10/300 GL column.

(F) SDS-PAGE of purified $TifA_{RB69}$-MBP-His_6 variants following purification of proteins from cell lysate through Ni-NTA resin.

Figure S7. Characterization of RNAs co-purifying with ToxN-TifA.

(A) Size-exclusion chromatogram of purified ToxIN run on a Superose 6 Increase 10/300 GL column.

(B) (*top*) Size-exclusion chromatogram of purified ToxN, *toxI*, and $TifA_{RB69}$-FLAG run on a Superose 6 Increase 10/300 GL column, with collected fractions indicated. (*bottom*) Anti-FLAG Western blot probing for $TifA_{RB69}$-FLAG in various FPLC fractions, indicating that $TifA_{RB69}$ localizes in the high molecular weight peak but does not colocalize with the ToxIN complex.

(C) Scatter plots comparing the summed RNA-seq counts (log_2 rpm) for individual genes in the *E. coli* BL21 genome in each set of two biological replicate samples (TifA WT sample eluent and lysate and TifA mutant sample eluent and lysate).

(D) Scatter plot comparing the mean summed RNA-seq counts (log_2 rpm) for individual genes in TifA WT sample whole cell lysate and chitin resin eluent (two biological replicates each). Genes with rpm values greater than or equal to 3 in all sequenced libraries (lysates and eluents) are shown.

(E) Scatter plot comparing the mean summed RNA-seq counts (log_2 rpm) for individual genes in TifA mutant sample whole cell lysate and chitin resin eluent (two biological replicates each). Genes with rpm values greater than or equal to 3 in all sequenced libraries (lysates and eluents) are shown.

(F) Western blot of ToxN-His_6 or ToxN(K55A)-His_6 in cell lysate and following co-immunoprecipitation with $TifA_{T4}$-FLAG (wild-type) in T4-infected cells producing $TifA_{T4}$ and ToxN.

Figure S1

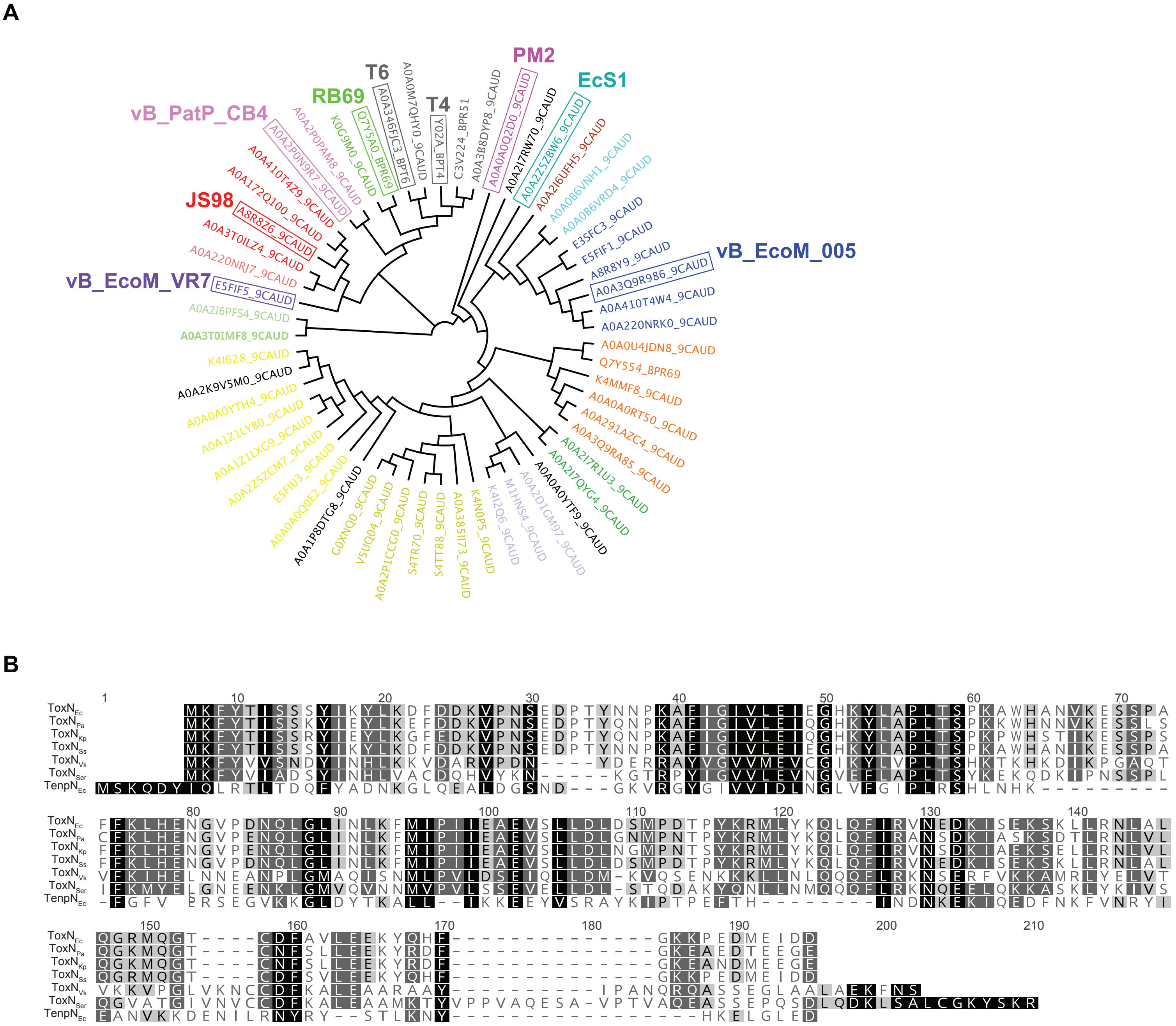
A
vB_PatP_CB4
RB69
T6
T4
PM2
EcS1
JS98
vB_EcoM_VR7
vB_EcoM_005
A0A2P0N9R7_9CAUD
A0A2P0PAM8_9CAUD
K0G9M0_9CAUD
Q7Y5A0_BPR69
A0A346FJC3_BPT6
A0A0M7QHY0_9CAUD
Y02A_BPT4
C3V224_BPR51
A0A3B8DYP8_9CAUD
A0A0A0Q2D0_9CAUD
A0A2I7RW70_9CAUD
A0A2Z5ZBW6_9CAUD
A0A2I6UFH5_9CAUD
A0A0B6VNH1_9CAUD
A0A0B6VRD4_9CAUD
E3SFC3_9CAUD
E5FIF1_9CAUD
A8R8Y9_9CAUD
A0A3Q9R986_9CAUD
A0A410T4W4_9CAUD
A0A220NRK0_9CAUD
A0A0U4JDN8_9CAUD
Q7Y554_BPR69
K4MMF8_9CAUD
A0A0A0RT50_9CAUD
A0A291AZC4_9CAUD
A0A3Q9RA85_9CAUD
A0A2I7R1U3_9CAUD
A0A2I7QYG4_9CAUD
A0A0A0YTF9_9CAUD
A0A2D1GM97_9CAUD
M1HNS4_9CAUD
K4I2Q6_9CAUD
K4N0P5_9CAUD
A0A385II73_9CAUD
S4TT88_9CAUD
S4TR70_9CAUD
A0A2P1CCG0_9CAUD
V5UQ04_9CAUD
G0XNQ0_9CAUD
A0A1P8DTG8_9CAUD
A0A0A0Q0E2_9CAUD
E5FIU3_9CAUD
A0A2Z5ZCM7_9CAUD
A0A1Z1LXG9_9CAUD
A0A1Z1LYB0_9CAUD
A0A0A0YTH4_9CAUD
A0A2K9V5M0_9CAUD
K4I628_9CAUD
A0A3T0IMF8_9CAUD
A0A2I6PFS4_9CAUD
E5FIF5_9CAUD
A0A220NRJ7_9CAUD
A0A3T0ILZ4_9CAUD
A8R8Z6_9CAUD
A0A172Q100_9CAUD
A0A410T4Z9_9CAUD
B
ToxN_Ec
ToxN_Pa
ToxN_Kp
ToxN_Ss
ToxN_Vk
ToxN_Ser
TenpN_Ec

Figure S2

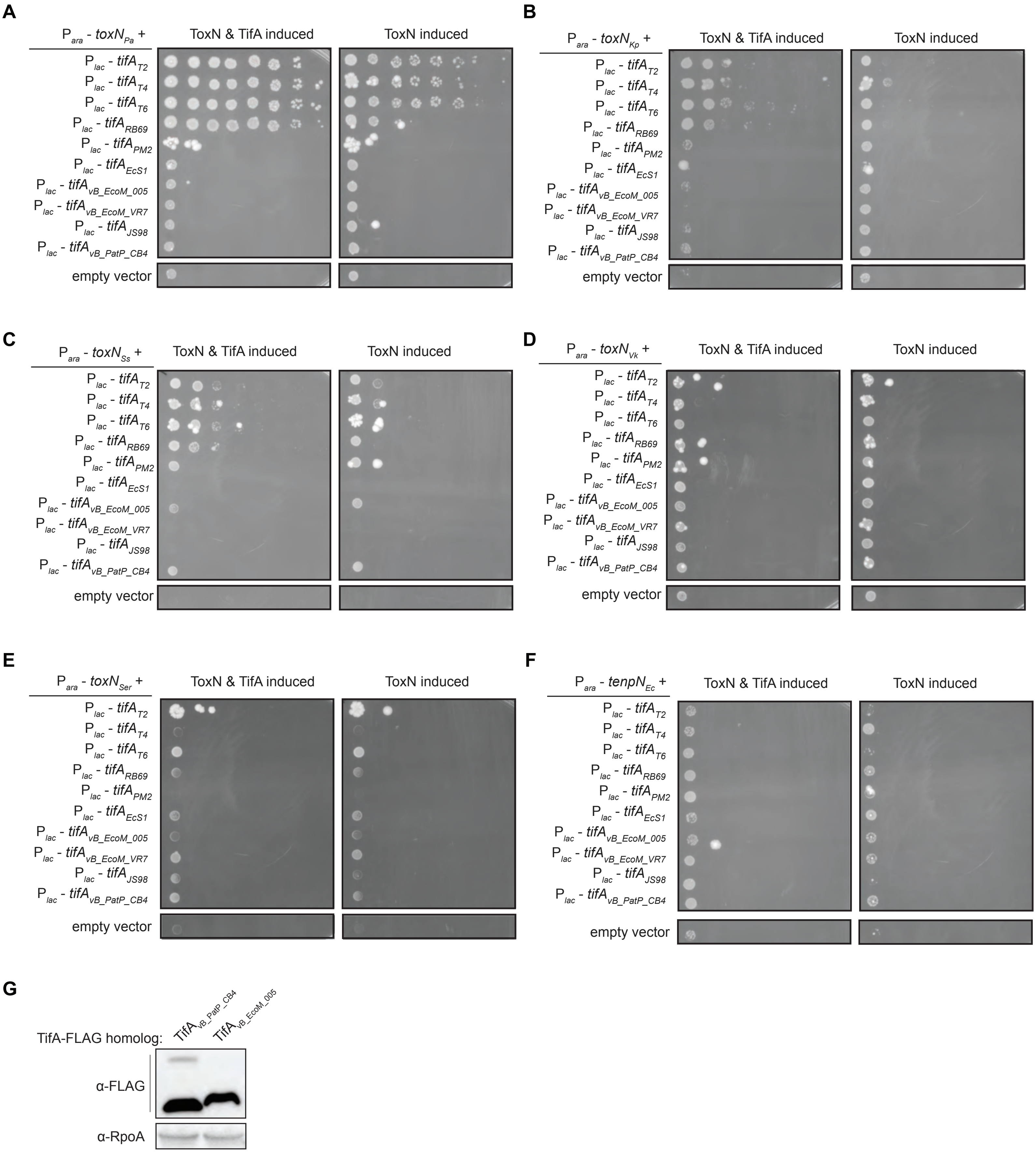
A
P_{ara} - $toxN_{Pa}$ +
ToxN & TifA induced
ToxN induced
P_{lac} - $tifA_{T2}$
P_{lac} - $tifA_{T4}$
P_{lac} - $tifA_{T6}$
P_{lac} - $tifA_{RB69}$
P_{lac} - $tifA_{PM2}$
P_{lac} - $tifA_{EcS1}$
P_{lac} - $tifA_{vB\_EcoM\_005}$
P_{lac} - $tifA_{vB\_EcoM\_VR7}$
P_{lac} - $tifA_{JS98}$
P_{lac} - $tifA_{vB\_PatP\_CB4}$
empty vector
B
P_{ara} - $toxN_{Kp}$ +
ToxN & TifA induced
ToxN induced
P_{lac} - $tifA_{T2}$
P_{lac} - $tifA_{T4}$
P_{lac} - $tifA_{T6}$
P_{lac} - $tifA_{RB69}$
P_{lac} - $tifA_{PM2}$
P_{lac} - $tifA_{EcS1}$
P_{lac} - $tifA_{vB\_EcoM\_005}$
P_{lac} - $tifA_{vB\_EcoM\_VR7}$
P_{lac} - $tifA_{JS98}$
P_{lac} - $tifA_{vB\_PatP\_CB4}$
empty vector
C
P_{ara} - $toxN_{Ss}$ +
ToxN & TifA induced
ToxN induced
P_{lac} - $tifA_{T2}$
P_{lac} - $tifA_{T4}$
P_{lac} - $tifA_{T6}$
P_{lac} - $tifA_{RB69}$
P_{lac} - $tifA_{PM2}$
P_{lac} - $tifA_{EcS1}$
P_{lac} - $tifA_{vB\_EcoM\_005}$
P_{lac} - $tifA_{vB\_EcoM\_VR7}$
P_{lac} - $tifA_{JS98}$
P_{lac} - $tifA_{vB\_PatP\_CB4}$
empty vector
D
P_{ara} - $toxN_{Vk}$ +
ToxN & TifA induced
ToxN induced
P_{lac} - $tifA_{T2}$
P_{lac} - $tifA_{T4}$
P_{lac} - $tifA_{T6}$
P_{lac} - $tifA_{RB69}$
P_{lac} - $tifA_{PM2}$
P_{lac} - $tifA_{EcS1}$
P_{lac} - $tifA_{vB\_EcoM\_005}$
P_{lac} - $tifA_{vB\_EcoM\_VR7}$
P_{lac} - $tifA_{JS98}$
P_{lac} - $tifA_{vB\_PatP\_CB4}$
empty vector
E
P_{ara} - $toxN_{Ser}$ +
ToxN & TifA induced
ToxN induced
P_{lac} - $tifA_{T2}$
P_{lac} - $tifA_{T4}$
P_{lac} - $tifA_{T6}$
P_{lac} - $tifA_{RB69}$
P_{lac} - $tifA_{PM2}$
P_{lac} - $tifA_{EcS1}$
P_{lac} - $tifA_{vB\_EcoM\_005}$
P_{lac} - $tifA_{vB\_EcoM\_VR7}$
P_{lac} - $tifA_{JS98}$
P_{lac} - $tifA_{vB\_PatP\_CB4}$
empty vector
F
P_{ara} - $tenpN_{Ec}$ +
ToxN & TifA induced
ToxN induced
P_{lac} - $tifA_{T2}$
P_{lac} - $tifA_{T4}$
P_{lac} - $tifA_{T6}$
P_{lac} - $tifA_{RB69}$
P_{lac} - $tifA_{PM2}$
P_{lac} - $tifA_{EcS1}$
P_{lac} - $tifA_{vB\_EcoM\_005}$
P_{lac} - $tifA_{vB\_EcoM\_VR7}$
P_{lac} - $tifA_{JS98}$
P_{lac} - $tifA_{vB\_PatP\_CB4}$
empty vector
G
TifA-FLAG homolog:
$TifA_{vB\_PatP\_CB4}$
$TifA_{vB\_EcoM\_005}$
α-FLAG
α-RpoA

Figure S3

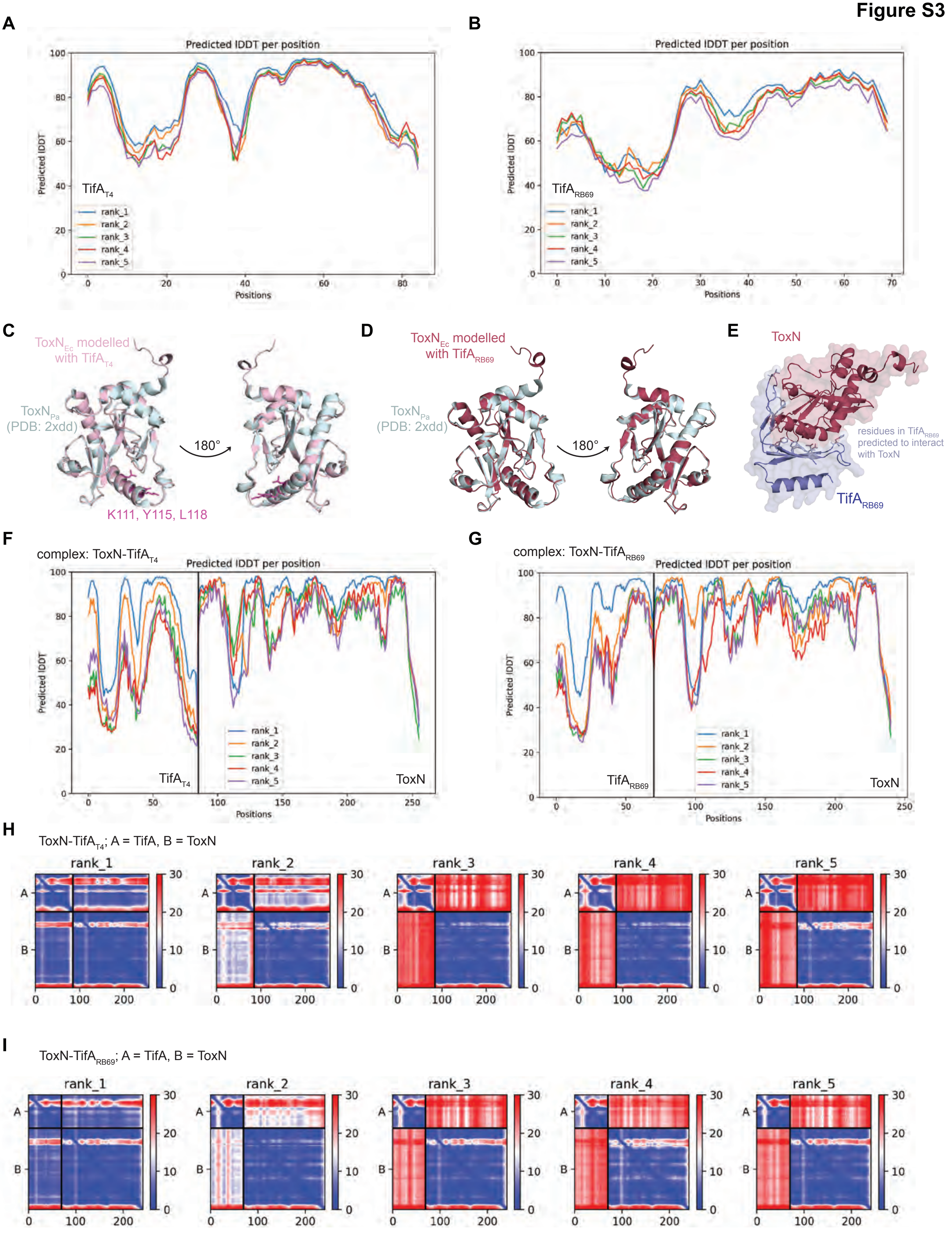
A
Predicted lDDT per position
Predicted lDDT
TifA$_{T4}$
rank_1
rank_2
rank_3
rank_4
rank_5
Positions
B
Predicted lDDT per position
Predicted lDDT
TifA$_{RB69}$
Positions
C
ToxN$_{Ec}$ modelled with TifA$_{T4}$
ToxN$_{Pa}$ (PDB: 2xdd)
180°
K111, Y115, L118
D
ToxN$_{Ec}$ modelled with TifA$_{RB69}$
ToxN$_{Pa}$ (PDB: 2xdd)
180°
E
ToxN
residues in TifA$_{RB69}$ predicted to interact with ToxN
TifA$_{RB69}$
F
complex: ToxN-TifA$_{T4}$
Predicted lDDT per position
TifA$_{T4}$
ToxN
Positions
G
complex: ToxN-TifA$_{RB69}$
Predicted lDDT per position
TifA$_{RB69}$
ToxN
Positions
H
ToxN-TifA$_{T4}$; A = TifA, B = ToxN
rank_1
rank_2
rank_3
rank_4
rank_5
I
ToxN-TifA$_{RB69}$; A = TifA, B = ToxN
rank_1
rank_2
rank_3
rank_4
rank_5

Figure S4

A

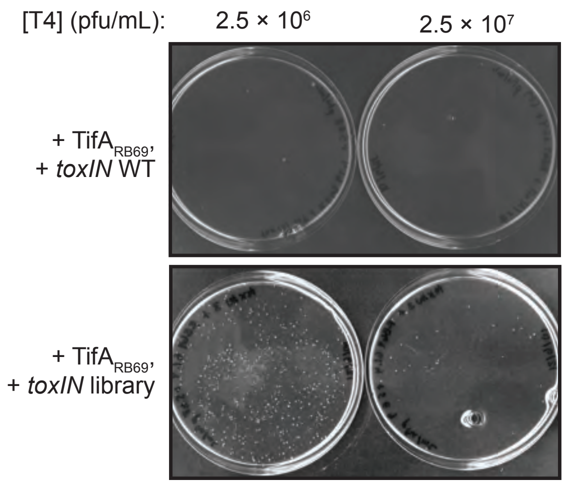
[T4] (pfu/mL):
2.5 × 10^6
2.5 × 10^7
+ TifA RB69,
+ toxIN WT
+ TifA RB69,
+ toxIN library

B

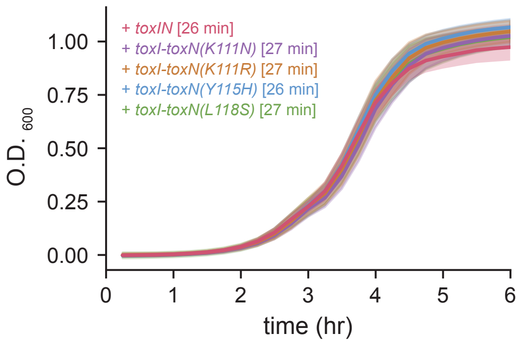
+ toxIN [26 min]
+ toxI-toxN(K111N) [27 min]
+ toxI-toxN(K111R) [27 min]
+ toxI-toxN(Y115H) [26 min]
+ toxI-toxN(L118S) [27 min]
O.D. 600
1.00
0.75
0.50
0.25
0.00
0
1
2
3
4
5
6
time (hr)

Figure S5

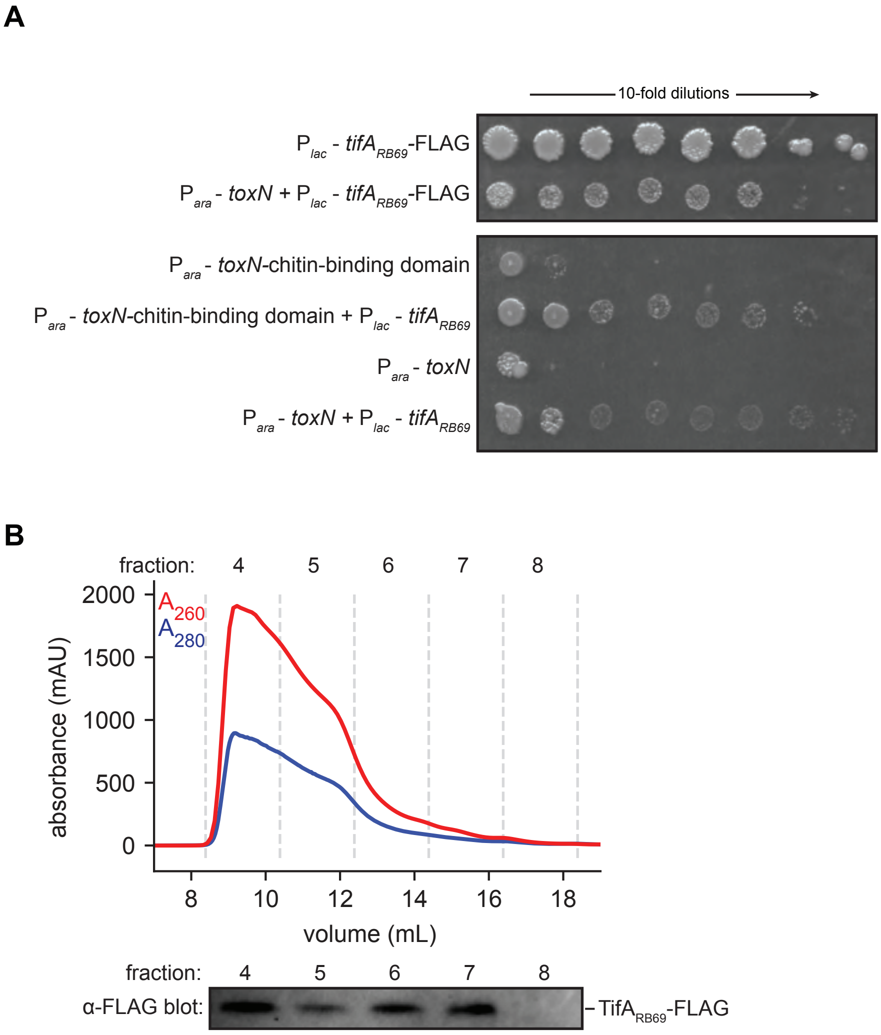

A
10-fold dilutions
P lac - tifA RB69-FLAG
P ara - toxN + P lac - tifA RB69-FLAG
P ara - toxN-chitin-binding domain
P ara - toxN-chitin-binding domain + P lac - tifA RB69
P ara - toxN
P ara - toxN + P lac - tifA RB69
B
fraction: 4 5 6 7 8
A260
A280
absorbance (mAU)
2000
1500
1000
500
0
8 10 12 14 16 18
volume (mL)
fraction: 4 5 6 7 8
α-FLAG blot:
TifA RB69-FLAG

Figure S6

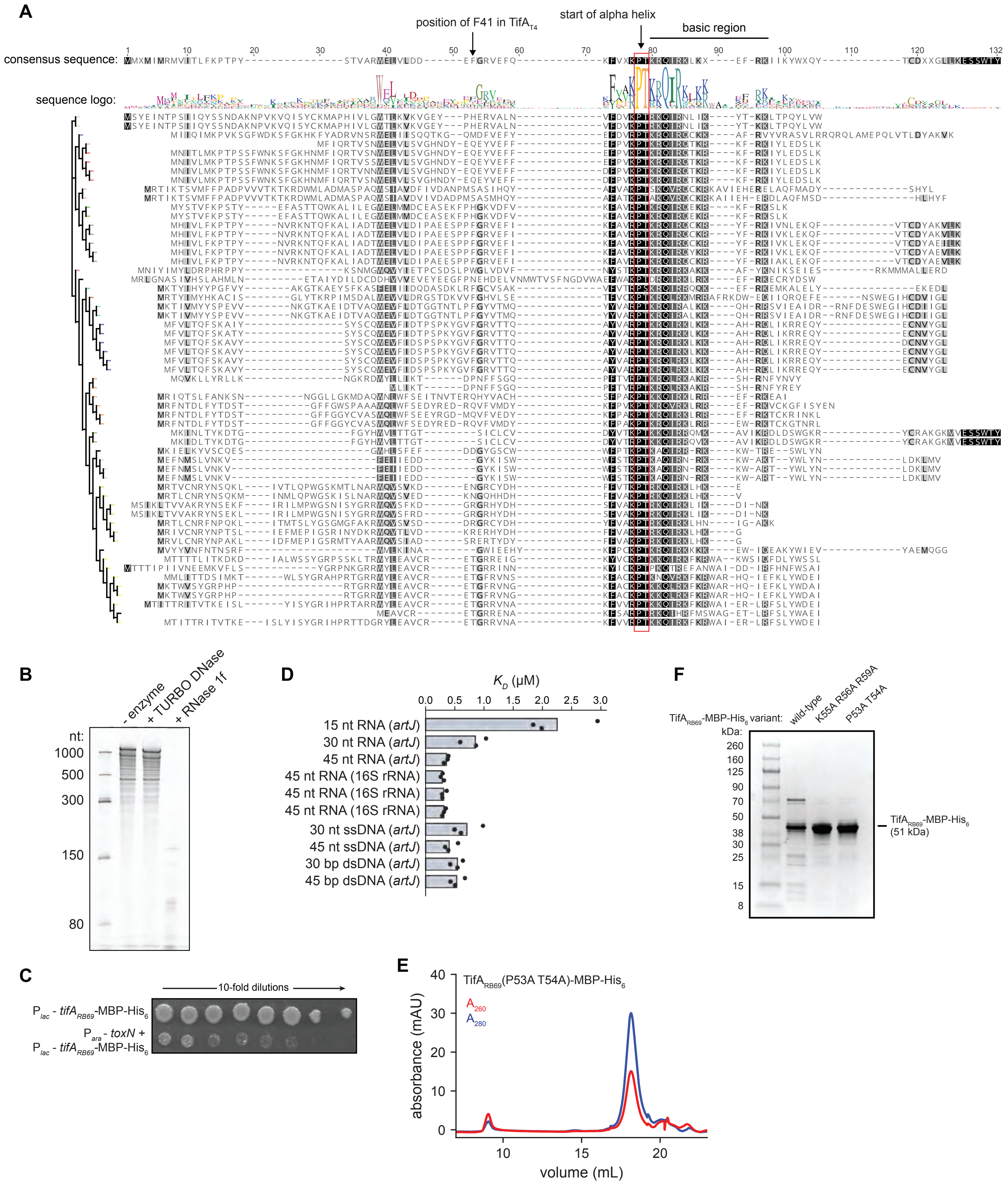

A
position of F41 in TifA T4
start of alpha helix
basic region
consensus sequence:
sequence logo:
B
- enzyme
+ TURBO DNase
+ RNase 1f
nt:
1000
500
300
150
80
C
10-fold dilutions
P lac - tifA RB69-MBP-His6
P ara - toxN +
P lac - tifA RB69-MBP-His6
D
KD (μM)
0.0 0.5 1.0 1.5 2.0 2.5 3.0
15 nt RNA (artJ)
30 nt RNA (artJ)
45 nt RNA (artJ)
45 nt RNA (16S rRNA)
45 nt RNA (16S rRNA)
45 nt RNA (16S rRNA)
30 nt ssDNA (artJ)
45 nt ssDNA (artJ)
30 bp dsDNA (artJ)
45 bp dsDNA (artJ)
E
TifA RB69(P53A T54A)-MBP-His6
A260
A280
absorbance (mAU)
volume (mL)
F
TifA RB69-MBP-His6 variant:
wild-type
K55A R56A R59A
P53A T54A
kDa:
260
160
125
90
70
50
38
30
25
15
8
TifA RB69-MBP-His6
(51 kDa)

Figure S7

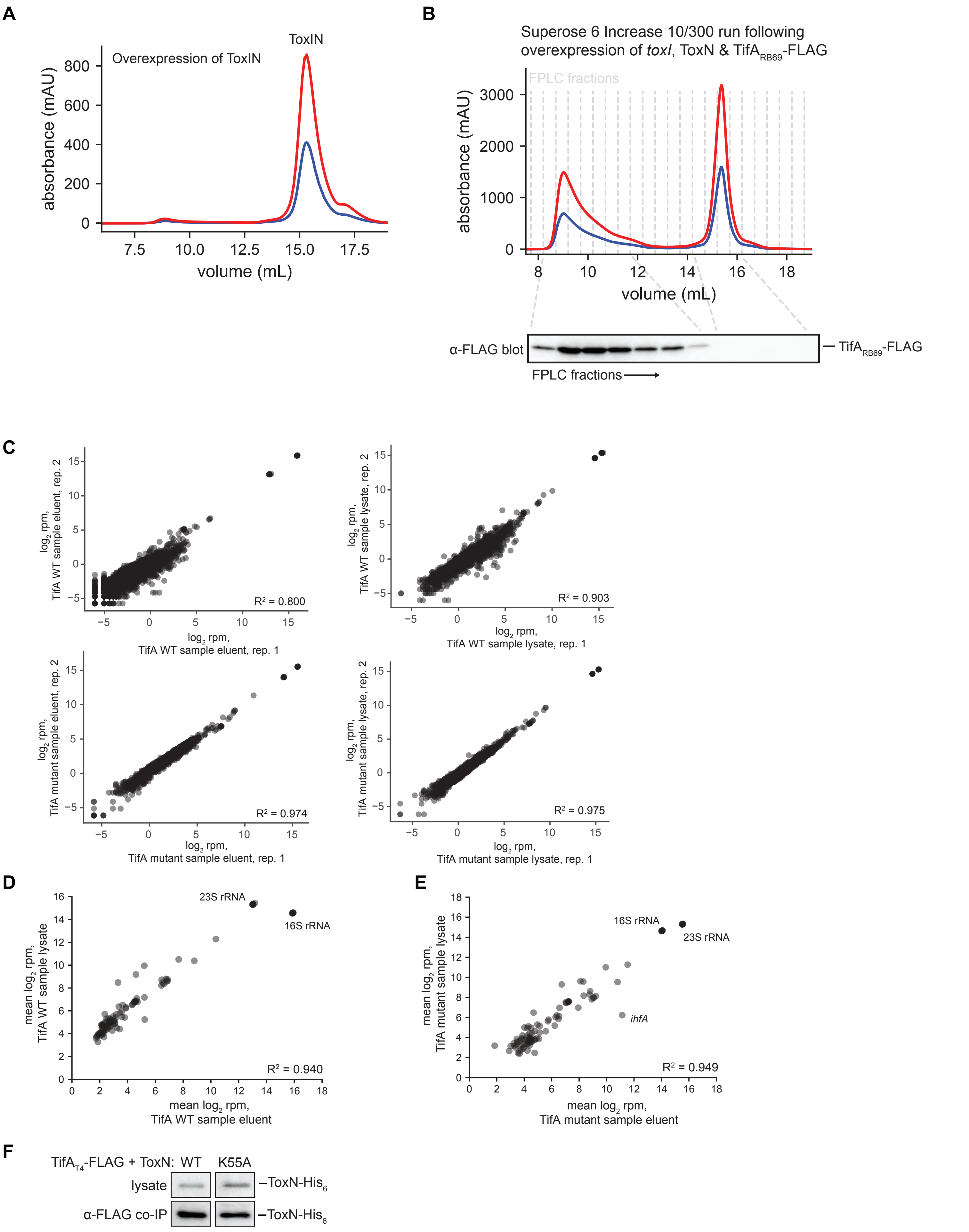
A
Overexpression of ToxIN
ToxIN
absorbance (mAU)
volume (mL)
B
Superose 6 Increase 10/300 run following overexpression of toxI, ToxN & TifA_RB69-FLAG
FPLC fractions
absorbance (mAU)
volume (mL)
α-FLAG blot
TifA_RB69-FLAG
FPLC fractions
C
log2 rpm, TifA WT sample eluent, rep. 2
log2 rpm, TifA WT sample eluent, rep. 1
R² = 0.800
log2 rpm, TifA WT sample lysate, rep. 2
log2 rpm, TifA WT sample lysate, rep. 1
R² = 0.903
log2 rpm, TifA mutant sample eluent, rep. 2
log2 rpm, TifA mutant sample eluent, rep. 1
R² = 0.974
log2 rpm, TifA mutant sample lysate, rep. 2
log2 rpm, TifA mutant sample lysate, rep. 1
R² = 0.975
D
23S rRNA
16S rRNA
mean log2 rpm, TifA WT sample lysate
mean log2 rpm, TifA WT sample eluent
R² = 0.940
E
16S rRNA
23S rRNA
ihfA
mean log2 rpm, TifA mutant sample lysate
mean log2 rpm, TifA mutant sample eluent
R² = 0.949
F
TifA_T4-FLAG + ToxN: WT K55A
lysate
α-FLAG co-IP
–ToxN-His6
–ToxN-His6

Table S1. Reagents, Assays, and Software

Critical commercial reagents, assays, and software used in this study.

Table S2. Protein sequences for ToxN, TenpN, and TifA homologs

Protein sequences for ToxN, TenpN, and TifA homologs used for the neutralization screen in Figure 1.

Table. S3. Strains

E. coli strains generated and used in this study.

Table S4. Plasmids

Plasmids generated and used in this study.

Table S5. Primers and Oligos

DNA and RNA oligos used in this study.

Table S6. G-blocks

G-block sequences used to clone ToxN and TifA homologs used for the neutralization screen in Figure 1.

Table S1. Reagents, Assays and Software

**Antibodies**		
6x-His epitope tag antibody (rabbit monoclonal)	Abcam	Cat #: AB200537
DYKDDDDK epitope tag antibody (rabbit monoclonal)	Cell Signaling Technology	Cat #: 14793
Purified anti-*E. coli* RNA Polymerase α antibody (mouse monoclonal)	BioLegend	Cat #: 663104
Anti-FLAG M2 antibody (mouse monoclonal)	Sigma	Cat #: F1804
Anti-FLAG M2 magnetic beads (mouse monoclonal)	Sigma	Cat #: 14793
Goat anti-rabbit IgG (H+L) secondary antibody, HRP (goat polyclonal)	ThermoFisher Scientific	Ca t#: 32460
**Bacterial and Virus Strains**		
For a complete list of bacterial strains used in this study, see Table S3.		
E. coli MG1655		ML6
E. coli DH5α	Invitrogen	Cat #: EC0112
E. coli TOP10	Invitrogen	Cat #: C404010
T7 Express Competent *E. coli* (High Efficiency)	NEB	Cat #: C2566
T4D	Gift from R. Young	phML-31
RB69	Félix d'Hérelle Reference Center for Bacterial Viruses, Université Laval	phML-39

T4 evo 3	Srikant *et al.*, 2022	N/A
T4 evo 2 (*tifA F41S*)	Srikant *et al.*, 2022	N/A
Thu1/R2	This study	N/A
**Chemicals, Peptides, and Recombinant Proteins**		
Carbenicillin	Gold Bio	Cat #: C-103
Chloramphenicol	Millipore Sigma	Cat #: C0378
Elution Buffer (EB)	QIAGEN	Cat #: 19086
GlycoBlue Coprecipitant (15 mg/mL)	Thermo Fisher	Cat #: AM9515
Kapa Biosystems HiFi Hotstart Readymix	Fisher Scientific	Cat #: NC0465187
Methanol	Millipore Sigma	Cat #: 179337
PEG 8000	Hampton Research	Cat #: HR2-515
Phenol-chloroform-isoamyl alcohol (25:24:1)	Millipore Sigma	Cat #: 77617-100ML
Second Strand Buffer	Thermo Fisher	Cat #: 10812014
SUPERase•In RNase Inhibitor (20 U/mL)	Thermo Fisher	Cat #: AM2694
SuperScript III Reverse Transcriptase	Thermo Fisher	Cat #: 18080044
SYBR Gold Nucleic Acid Gel Stain (10,000X Concentrate in DMSO)	Thermo Fisher	Cat #: S11494
HpaI	NEB	Cat #: R0105
T4 DNA Ligase Reaction Buffer	NEB	Cat #: B0202
T4 DNA Ligase	NEB	Cat #: M0202

T4 Polynucleotide Kinase	NEB	Cat #: M0201
T4 DNA Polymerase	NEB	Cat #: M0203
Isopropyl-D-1-thiogalactopyranoside	Goldbio	Cat #: I2481C
L-arabinose	Fisher Scientific	Cat #: AC365185000
Anhydrotetracycline hydrochloride	Sigma-Aldrich	Cat #: 37919-100MG-R
2x Gibson Assembly Master Mix	NEB	Cat #: E2611
2x Novex TBE-Urea sample buffer	Invitrogen	Cat #: LC6876
Phenol solution saturated with 0.1 M ctrate buffer, pH = 4.3 ± 0.2	Sigma-Aldrich	Cat #: P4682
Acid-Phenol:Chloroform, pH 4.5 (with IAA, 125:24:1)	Thermo Fisher	Cat #: AM9720
Sodium Acetate (3 M), pH 5.5, RNAse-free	Thermo Fisher	Cat #: AM9740
DEPC-treated water	Invitrogen	Cat #: AM9906
NdeI	NEB	Cat #: R0111
SapI	NEB	Cat #: R0569
AvrII	NEB	Cat #: R0174
SacI-HF	NEB	Cat #: R3156L
SalI-HF	NEB	Cat #: R3138L
10x CutSmart buffer	NEB	Cat #: B7204S
SIGMA*FAST* protease inhibitor cocktail tablets, EDTA-free	Sigma-Aldrich	Cat #: S8830

Chitin resin	NEB	Cat #: S6651S
UltraPure BSA (50 mg/mL)	Invitrogen	Cat #: AM2616
**Critical Commercial Assays, Kits, and Equipment**		
SuperSignal West Femto Maximum Sensitivity Substrate	Thermo Fisher	Cat #: 34095
ZR Plasmid Miniprep - Classic	Zymo	Cat #: D4016
Zymoclean Gel DNA Recovery Kit	Zymo	Cat #: D4001
Low Range ssRNA Ladder	NEB	Cat #: N0364S
Chameleon Duo Pre-Stained Protein Ladder	Licor	Cat #: 928-60000
Chitin resin	NEB	Cat #: S6651S
NEBNext Ultra II RNA Library Prep Kit	NEB	Cat #: E7770
Novex 6% TBE-Urea gel	Invitrogen	Cat #: EC6865BOX
4-20% Mini-PROTEAN TGX gels	Biorad	Cat #: 4561093
Superose 6 Increase 10/300 GL size exclusion column	Cytiva	Cat #: GE29-0915-96
**Deposited Data**		
RNA-Sequencing	This study	GSE234211
**Recombinant DNA**		

For a complete list of plasmids and G-blocks generated for this study, see Table S4.		
**Oligonucleotides**		
For a complete list of oligonucleotides used for this study, see Table S5.		
**Software and Algorithms**		
ImageJ (v1.48)	NIH	https://imagej.nih.gov/ij/
bowtie2 (v2.1.0)	Langmead and Salzberg, 2012	http://bowtie-bio.sourceforge.net/bowtie2/index.shtml
pysam (v0.9.1.4)	N/A	https://github.com/pysam-developers/pysam
samtools (v0.1.19)	Li et al., 2009	http://samtools.sourceforge.net/
numpy (v1.13.1)	N/A	https://github.com/numpy/numpy
biopython (v1.65)	N/A	https://github.com/biopython/biopython
scipy (v0.18.1)	N/A	https://github.com/scipy/scipy
jupyter notebook	N/A	https://github.com/jupyter
AlphaFold2	Jumper et al., 2021	https://colab.research.google.com/github/sokrypton/ColabFold/blob/main/AlphaFold2.ipynb
PYMOL 2.1	Schrodinger LLC	https://pymol.org/2/

Geneious Prime	Dotmatics	https://www.geneious.com/
Prism 9	GraphPad	https://www.graphpad.com/
Curveball	Ram et al., 2019	https://curveball.yoavram.com/

Table S2. Protein sequences for ToxN, TenpN, and TifA homologs

**Name**	**Description**	**Sequence (N→C-term)**
toxN$_{Ec}$	*toxN* from *E. coli* GCA_001012275	MKFYTISSSYIKYLKDFDDKVPNSEDPTYNNPKAFIGIV<br>LEIEGHKYLAPLTSPKAWHANVKESSPAFFKLHENGVP<br>DNQLGLINLKFMIPIIEAEVSLLDLDSMPDTPYKRMLYK<br>QLQFIRVNEDKISEKSKLLRNLALQGRMQGTCDFAVLE<br>EKYQHFGKKPEDMEIDD
toxN$_{Kp}$	*toxN* from *K. pneumoniae* (accession number A0A331BKA2_KLEPN)	MKFYTISSRYIEYLKGFEDKVPNSEDPTYQNPKAFIGIV<br>LEIQGHKYLAPLTSPKPWHSTIKESSPSFFKLHENGVPE<br>NQLGLINLKFMIPIIEAEVSLLDLNGMPNTSYKRMLYK<br>QLQFIRVNSDKIAEKSELLRNLVLQGKMQGTCNFSLLE<br>EKYRDFGKEANDMEEGE
tenpN$_{Ec}$	*tenpN* from *E. coli* plasmid pWP4-S17-ESBL-08_3 (accession number A0A4T8VJ30_ECOLX)	MSKQDYIQLRTLTDQFYADNKGLQEALDGSNDGKVR<br>GYGIVVIDLNGLVFGIPLRSHLNHKFGFVSERSEGVKK<br>GLDYTKALLIKKEEYVSRAYKIPTPEFTHINDNKEKIQE<br>DFNKFVNRYIEANVKKDENILRNYRYSTLKNYHKELG<br>LED
toxN$_{Ss}$	*toxN* from *S. sonnei* (accession number A0A3U1U8P6_SHISO)	MKFYTISSSYIKYLKDFDDKVPNSEDPTYNNPKAFIGIV<br>LEIEGHKYLAPLTSPKAWHANIKESSPAFFKLHENGVP<br>DNQLGLINLKFMIPIIEAEVSLLDLDSMPDTPYKRMLYK<br>QLQFIRVNEDKISEKSKLLRNLALQGRMQGTCDFSVLE<br>EKYQHFGKKPEDMEIDD
toxN$_{Vk}$	*toxN* from *V. kanaloae* (accession number A0A2N7J7F4_9VIBR)	MKFYVVSNDYINHLKKVDARVPDNYDERRAYVGVV<br>MEVCGIKYLVPLTSHKTKHKDIKPGAQTVFKIHELNNE<br>ANPLGMAQISNMLPVLDSEIQLLDMKVQSENKKKLLN<br>LQQQFLRKNSERFVKKAMRLYELVTVKKVPGLVKNC<br>CDFKALEAARAAYIPANQRQASSEGLAALAEKFNS
toxN$_{Ser.}$	*toxN* from *Serratia* sp. SRS-8-S-2018 (accession number A0A506VJT7_9GAMM)	MKFYVIADSYINHLVACDQHVYKNKGTRPYIGVVLEV<br>NGVEFLAPLTSYKEKQDKIPNSSPLIFKMYELGNEENK<br>LGMVQVNNMVPVLSSEVELLDLSTQDAKYQNLLNMQ<br>QQFLRKNQEELQKKASKLYKIVSQGVATGIVNVCCDF

		KALEAAMKTYVPPVAQESAVPTVAQEASSEPQSDLQDKLSALCGKYSKR
toxN$_{Pa}$	*toxN* from *P. atrosepticum* plasmid pECA1039	MKFYTISSKYIEYLKEFDDKVPNSEDPTYQNPKAFIGIVLEIQGHKYLAPLTSPKKWHNNVKESSLSCFKLHENGVPENQLGLINLKFMIPIIEAEVSLLDLGNMPNTPYKRMLYKQLQFIRANSDKIASKSDTLRNLVLQGKMQGTCNFSLLEEKYRDFGKEAEDTEEGE
tifA$_{T2}$	*tifA* from *Escherichia* phage T2	MHIVLFKPTPYNVRKNTQFKALIADTWELVLDIPAEESPPFGRVEFIKFAVRPTKRQIRQCKRYFRKIVKLEKQFVTCDYAKVLK
tifA$_{T4}$	*tifA* from *Escherichia* phage T4 (NCBI: NC_0008_66.4)	MHIVLFKPTPYNVRKNTQFKALIADTWELVLDIPAEESPPFGRVEFIKFAVRPTKRQIRQCKRYFRKIVKLEKQFVTCDYAEILK
tifA$_{T6}$	*tifA* from *Escherichia* phage T6 (EMBL-EBI:AP018814)	MHIVLFKPTPYNVRKNTQFKALIADTWELVLDIPAEESPPFGRVEFIKFAVRPTKRQIRQCKRYFRKIVNLEKQFVTCDYAKVLK
tifA$_{RB69}$	*tifA* from *Escherichia* phage RB69 (NCBI:NC_004928.1)	MYSTVFKPSTYEFASTTQWKALILEGWELMMDCEASEKFPHGKVDFVKFAVRPTKRQIRQEKRKFRKSLK
tifA$_{EcS1}$	*tifA* from *Escherichia* phage EcS1 (EMBL-EBI: LC371242)	MKTYIHYYPGFVYAKGTKAEYSFKASFELIIDQDASDKLRFGCVSAKVFVTRPSKRQIRRQQKEFRKEMKALELYEKEDL
tifA$_{vB\_EcoM\_005}$	*tifA* from *Escherichia* phage vB_EcoM_005 (EMBL-EBI:MK295203)	MFVLTQFSKAVYSYSCQWEVFIDSPSPKYGVFGRVTTQAYVARPTKRQIRKLKKAHRQLIKRREQYECNVYGL
tifA$_{vB\_EcoM\_VR7}$	*tifA* from *Escherichia* phage vB_EcoM_VR7 (NCBI:NC_014792.1)	MIIQIMKPVKSGFWDKSFGKHKFYADRVNSRWELIISQQTKGQMDFVEFYEFDVRPTKRQIRQCKKAFRVYVRASVLRRQRQLAMEPQLVTLDYAKVK
tifA$_{JS98}$	*tifA* from *Escherichia* phage JS98 (NCBI:NC_010105.1)	MNIVLMKPTPSSFWNKSFGKHNMFIQRTVSSWELVLSVGHNDYEQEYVEFFEFPVKPTKRQIRQTKRKFRKIYLEDSLK

tifA$_{PM2}$	*tifA* from *Pectobacterium* phage PM2 (NCBI: NC_028940.1)	MNIYIMYLDRPHRPPYKSNMGWQVYIETPCSDSLPWGLVDVFNYSTKPTKRQIRKAKKAFYKNIKSEIESRKMMMALLERD
tifA$_{vB\_PatP\_CB4}$	*tifA* from *Pectobacterium* phage vB_PatP_CB4 (EMBL-EBI:KY549659)	MRTIKTSVMFFPADPVVVTKTKRDWMLADMASPAQWSIAVDFIVDANPMSASIHQYAFVAKPTSKQVRQCKRKAVIEHERELAQFMADYSHYL

Table S3. Strains

Name	Genotype	Source
ML6	MG1655	
DH5α	Cloning strain	Invitrogen
TOP10	Cloning strain	
ML3326	MG1655 pBAD33-*toxN* pEXT20-*toxI*	Guegler and Laub, 2021
ML3778	MG1655 pBAD33-*toxN* pEXT20-$tifA_{T2}$	Srikant, Guegler, and Laub, 2022
ML3779	MG1655 pBAD33-*toxN* pEXT20-$tifA_{T4}$	Srikant, Guegler, and Laub, 2022
ML3780	MG1655 pBAD33-*toxN* pEXT20-$tifA_{T6}$	Srikant, Guegler, and Laub, 2022
ML3781	MG1655 pBAD33-*toxN* pEXT20-$tifA_{RB69}$	Srikant, Guegler, and Laub, 2022
ML4188	MG1655 pEXT20-$tifA_{vB\_PatP\_CB4}$-FLAG	This study
ML4195	MG1655 pEXT20-$tifA_{vB\_EcoM\_005}$-FLAG	This study
gCKG-517	MG1655 pBAD33-*toxN* pEXT20- $tifA_{RB69}$*(P53A T54A)*	This study
gCKG-646	MG1655 pBAD33-*toxN* pEXT20- $tifA_{RB69}$*(K55A R56A R59A)*	This study
ML4118	MG1655 pBAD33-*toxN(K111N)* pEXT20-$tifA_{T4}$	This study
ML4119	MG1655 pBAD33-*toxN(K111R)* pEXT20-$tifA_{T4}$	This study
ML4120	MG1655 pBAD33-*toxN(L118S)* pEXT20-$tifA_{T4}$	This study
ML4121	MG1655 pBAD33-*toxN(Y115H)* pEXT20-$tifA_{T4}$	This study
ML4122	MG1655 pBAD33-*toxN(K111N)* pEXT20-$tifA_{RB69}$	This study
ML4123	MG1655 pBAD33-*toxN(K111R)* pEXT20-$tifA_{RB69}$	This study
ML4124	MG1655 pBAD33-*toxN(L118S)* pEXT20-$tifA_{RB69}$	This study

ML4125	MG1655 pBAD33-*toxN(Y115H)* pEXT20-$tifA_{RB69}$	This study
ML4126	MG1655 pBAD33-*toxN(K111N)* pEXT20-*toxI*	This study
ML4127	MG1655 pBAD33-*toxN(K111R)* pEXT20-*toxI*	This study
ML4128	MG1655 pBAD33-*toxN(L118S)* pEXT20-*toxI*	This study
ML4129	MG1655 pBAD33-*toxN(Y115H)* pEXT20-*toxI*	This study
gCKG-472	MG1655 pBAD33-$toxN_{Ec}$ pEXT20-$tifA_{EcS1}$	This study
gCKG-473	MG1655 pBAD33-$toxN_{Ec}$ pEXT20-$tifA_{vB\_EcoM\_005}$	This study
gCKG-568	MG1655 pBAD33-$toxN_{Ec}$ pEXT20-$tifA_{vB\_EcoM\_VR7}$	This study
gCKG-474	MG1655 pBAD33-$toxN_{Ec}$ pEXT20-$tifA_{JS98}$	This study
gCKG-471	MG1655 pBAD33-$toxN_{Ec}$ pEXT20-$tifA_{PM2}$	This study
gCKG-475	MG1655 pBAD33-$toxN_{Ec}$ pEXT20-$tifA_{vB\_PatP\_CB4}$	This study
gCKG-252	MG1655 pBAD33-$toxN_{Pa}$ pEXT20-$tifA_{T2}$	This study
gCKG-253	MG1655 pBAD33-$toxN_{Pa}$ pEXT20-$tifA_{T4}$	This study
gCKG-254	MG1655 pBAD33-$toxN_{Pa}$ pEXT20-$tifA_{T6}$	This study
gCKG-255	MG1655 pBAD33-$toxN_{Pa}$ pEXT20-$tifA_{RB69}$	This study
gCKG-552	MG1655 pBAD33-$toxN_{Pa}$ pEXT20-$tifA_{EcS1}$	This study
gCKG-560	MG1655 pBAD33-$toxN_{Pa}$ pEXT20-$tifA_{EcoM\_005}$	This study
gCKG-569	MG1655 pBAD33-$toxN_{Pa}$ pEXT20-$tifA_{EcoM\_VR7}$	This study
gCKG-577	MG1655 pBAD33-$toxN_{Pa}$ pEXT20-$tifA_{JS98}$	This study
gCKG-257	MG1655 pBAD33-$toxN_{Pa}$ pEXT20-$tifA_{PM2}$	This study
gCKG-258	MG1655 pBAD33-$toxN_{Pa}$ pEXT20-$tifA_{PatP\_CB4}$	This study

gCKG-521	MG1655 pBAD33-*toxN*$_{Kp}$ pEXT20-*tifA*$_{T2}$	This study
gCKG-528	MG1655 pBAD33-*toxN*$_{Kp}$ pEXT20-*tifA*$_{T4}$	This study
gCKG-534	MG1655 pBAD33-*toxN*$_{Kp}$ pEXT20-*tifA*$_{T6}$	This study
gCKG-540	MG1655 pBAD33-*toxN*$_{Kp}$ pEXT20-*tifA*$_{RB69}$	This study
gCKG-555	MG1655 pBAD33-*toxN*$_{Kp}$ pEXT20-*tifA*$_{EcS1}$	This study
gCKG-563	MG1655 pBAD33-*toxN*$_{Kp}$ pEXT20-*tifA*$_{vB\_EcoM\_005}$	This study
gCKG-572	MG1655 pBAD33-*toxN*$_{Kp}$ pEXT20-*tifA*$_{vB\_EcoM\_VR7}$	This study
gCKG-580	MG1655 pBAD33-*toxN*$_{Kp}$ pEXT20-*tifA*$_{JS98}$	This study
gCKG-547	MG1655 pBAD33-*toxN*$_{Kp}$ pEXT20-*tifA*$_{PM2}$	This study
gCKG-587	MG1655 pBAD33-*toxN*$_{Kp}$ pEXT20-*tifA*$_{vB\_PatP\_CB4}$	This study
gCKG-524	MG1655 pBAD33-*toxN*$_{Ss}$ pEXT20-*tifA*$_{T2}$	This study
gCKG-530	MG1655 pBAD33-*toxN*$_{Ss}$ pEXT20-*tifA*$_{T4}$	This study
gCKG-536	MG1655 pBAD33-*toxN*$_{Ss}$ pEXT20-*tifA*$_{T6}$	This study
gCKG-542	MG1655 pBAD33-*toxN*$_{Ss}$ pEXT20-*tifA*$_{RB69}$	This study
gCKG-557	MG1655 pBAD33-*toxN*$_{Ss}$ pEXT20-*tifA*$_{EcS1}$	This study
gCKG-565	MG1655 pBAD33-*toxN*$_{Ss}$ pEXT20-*tifA*$_{vB\_EcoM\_005}$	This study
gCKG-574	MG1655 pBAD33-*toxN*$_{Ss}$ pEXT20-*tifA*$_{vB\_EcoM\_VR7}$	This study
gCKG-582	MG1655 pBAD33-*toxN*$_{Ss}$ pEXT20-*tifA*$_{JS98}$	This study
gCKG-549	MG1655 pBAD33-*toxN*$_{Ss}$ pEXT20-*tifA*$_{PM2}$	This study
gCKG-589	MG1655 pBAD33-*toxN*$_{Ss}$ pEXT20-*tifA*$_{vB\_PatP\_CB4}$	This study
gCKG-525	MG1655 pBAD33-*toxN*$_{Vk}$ pEXT20-*tifA*$_{T2}$	This study

gCKG-531	MG1655 pBAD33-*toxN*$_{Vk}$ pEXT20-*tifA*$_{T4}$	This study
gCKG-537	MG1655 pBAD33-*toxN*$_{Vk}$ pEXT20-*tifA*$_{T6}$	This study
gCKG-543	MG1655 pBAD33-*toxN*$_{Vk}$ pEXT20-*tifA*$_{RB69}$	This study
gCKG-558	MG1655 pBAD33-*toxN*$_{Vk}$ pEXT20-*tifA*$_{EcS1}$	This study
gCKG-566	MG1655 pBAD33-*toxN*$_{Vk}$ pEXT20-*tifA*$_{vB\_EcoM\_005}$	This study
gCKG-575	MG1655 pBAD33-*toxN*$_{Vk}$ pEXT20-*tifA*$_{vB\_EcoM\_VR7}$	This study
gCKG-583	MG1655 pBAD33-*toxN*$_{Vk}$ pEXT20-*tifA*$_{JS98}$	This study
gCKG-550	MG1655 pBAD33-*toxN*$_{Vk}$ pEXT20-*tifA*$_{PM2}$	This study
gCKG-590	MG1655 pBAD33-*toxN*$_{Vk}$ pEXT20-*tifA*$_{vB\_PatP\_CB4}$	This study
gCKG-526	MG1655 pBAD33-*toxN*$_{Ser.}$ pEXT20-*tifA*$_{T2}$	This study
gCKG-532	MG1655 pBAD33-*toxN*$_{Ser.}$ pEXT20-*tifA*$_{T4}$	This study
gCKG-538	MG1655 pBAD33-*toxN*$_{Ser.}$ pEXT20-*tifA*$_{T6}$	This study
gCKG-544	MG1655 pBAD33-*toxN*$_{Ser.}$ pEXT20-*tifA*$_{RB69}$	This study
gCKG-559	MG1655 pBAD33-*toxN*$_{Ser.}$ pEXT20-*tifA*$_{EcS1}$	This study
gCKG-567	MG1655 pBAD33-*toxN*$_{Ser.}$ pEXT20-*tifA*$_{vB\_EcoM\_005}$	This study
gCKG-576	MG1655 pBAD33-*toxN*$_{Ser.}$ pEXT20-*tifA*$_{vB\_EcoM\_VR7}$	This study
gCKG-584	MG1655 pBAD33-*toxN*$_{Ser.}$ pEXT20-*tifA*$_{JS98}$	This study
gCKG-551	MG1655 pBAD33-*toxN*$_{Ser.}$ pEXT20-*tifA*$_{PM2}$	This study
gCKG-591	MG1655 pBAD33-*toxN*$_{Ser.}$ pEXT20-*tifA*$_{vB\_PatP\_CB4}$	This study
gCKG-523	MG1655 pBAD33-*tenpN*$_{Ec}$ pEXT20-*tifA*$_{T2}$	This study
gCKG-529	MG1655 pBAD33-*tenpN*$_{Ec}$ pEXT20-*tifA*$_{T4}$	This study

gCKG-535	MG1655 pBAD33-*tenpN*$_{Ec}$ pEXT20-*tifA*$_{T6}$	This study
gCKG-541	MG1655 pBAD33-*tenpN*$_{Ec}$ pEXT20-*tifA*$_{RB69}$	This study
gCKG-556	MG1655 pBAD33-*tenpN*$_{Ec}$ pEXT20-*tifA*$_{EcS1}$	This study
gCKG-564	MG1655 pBAD33-*tenpN*$_{Ec}$ pEXT20-*tifA*$_{vB\_EcoM\_005}$	This study
gCKG-573	MG1655 pBAD33-*tenpN*$_{Ec}$ pEXT20-*tifA*$_{vB\_EcoM\_VR7}$	This study
gCKG-581	MG1655 pBAD33-*tenpN*$_{Ec}$ pEXT20-*tifA*$_{JS98}$	This study
gCKG-548	MG1655 pBAD33-*tenpN*$_{Ec}$ pEXT20-*tifA*$_{PM2}$	This study
gCKG-588	MG1655 pBAD33-*tenpN*$_{Ec}$ pEXT20-*tifA*$_{vB\_PatP\_CB4}$	This study
ML4197	MG1655 pBAD33-*toxN*-chitin binding domain pEXT20	This study
ML4198	MG1655 pBAD33-*toxN*-chitin binding domain pEXT20-*tifA*$_{RB69}$	This study
ML4189	MG1655 pBAD33 pEXT20-*tifA*$_{RB69}$-FLAG	This study
ML4190	MG1655 pBAD33-*toxN* pEXT20-*tifA*$_{RB69}$-FLAG	This study
ML4192	MG1655 pBAD33 pEXT20-*tifA*$_{RB69}$-MBP-His_6	This study
ML4193	MG1655 pBAD33-*toxN* pEXT20-*tifA*$_{RB69}$-MBP-His_6	This study
ML3328	MG1655 pBR322-*toxIN*	Guegler and Laub, 2021
ML4130	MG1655 pBR322-*toxI-toxN(L118S)*	This study
ML4131	MG1655 pBR322-*toxI-toxN(Y115H)*	This study
ML4132	MG1655 pBR322-*toxI-toxN(K111N)*	This study
ML4133	MG1655 pBR322-*toxI-toxN(K111R)*	This study
ML4134	MG1655 pBR322-*toxI-toxN(L118S)* pKVS45-*tifA*$_{T4}$	This study
ML4135	MG1655 pBR322-*toxI-toxN(Y115H)* pKVS45-*tifA*$_{T4}$	This study

ML4136	MG1655 pBR322-*toxI-toxN(K111N)* pKVS45-*tifA*$_{T4}$	This study
ML4137	MG1655 pBR322-*toxI-toxN(K111R)* pKVS45-*tifA*$_{T4}$	This study
ML4138	MG1655 pBR322-*toxI-toxN(L118S)* pKVS45-*tifA*$_{RB69}$	This study
ML4139	MG1655 pBR322-*toxI-toxN(Y115H)* pKVS45-*tifA*$_{RB69}$	This study
ML4140	MG1655 pBR322-*toxI-toxN(K111N)* pKVS45-*tifA*$_{RB69}$	This study
ML4141	MG1655 pBR322-*toxI-toxN(K111R)* pKVS45-*tifA*$_{RB69}$	This study
ML4142	MG1655 pBR322-*toxI-toxN(L118S)* pKVS45	This study
ML4143	MG1655 pBR322-*toxI-toxN(Y115H)* pKVS45	This study
ML4144	MG1655 pBR322-*toxI-toxN(K111N)* pKVS45	This study
ML4145	MG1655 pBR322-*toxI-toxN(K111R)* pKVS45	This study
ML3774	MG1655 pBR322-*toxIN* pKVS45-*tifA*$_{T4}$	Srikant, Guegler, and Laub, 2022
ML3786	MG1655 pBR322-*toxI-toxN*-His$_6$ pKVS45-*tifA*$_{T4}$-FLAG	Srikant, Guegler, and Laub, 2022
ML4171	MG1655 pBR322-*toxI-toxN*-His$_6$ pKVS45-*tifA*$_{T4}$*(P53A T54A)*-FLAG	This study
ML4172	MG1655 pBR322-*toxI-toxN*-His$_6$ pKVS45-*tifA*$_{T4}$*(K55A R56A R59A)*-FLAG	This study
ML4156	MG1655 pBR322-*toxI-toxN*-His$_6$ pKVS45-*tifA*$_{T4}$*(F41S)*-FLAG	This study
ML4157	MG1655 pBR322-*toxI-toxN*-His$_6$ pKVS45-*tifA*$_{T4}$*(F41H)*-FLAG	This study
ML4185	MG1655 pBR322-*toxI-toxN(K55A)*-His$_6$ pKVS45-*tifA*$_{T4}$-FLAG	This study
ML4158	T7 express pTXB1-*toxN* pACYC-*tifA*$_{RB69}$	This study

ML4159	T7 express pTXB1-*toxN* pACYC-*tifA*$_{RB69}$-FLAG	This study
ML4177	T7 express pTXB1-*toxN* pACYC Duet-*toxI-tifA*$_{RB69}$	This study
ML4163	T7 express pET-*tifA*$_{RB69}$-MBP-His$_6$	This study; parental vector from Addgene (Plasmid #37237)
ML4164	T7 express pET-*tifA*$_{RB69}$*(P53A T54A)*-MBP-His$_6$	This study; parental vector from Addgene (Plasmid #37237)
ML4165	T7 express pET-*tifA*$_{RB69}$*(K55A R56A R59A)*-MBP-His$_6$	This study; parental vector from Addgene (Plasmid #37237)
ML3343	DH5α pBAD33-*toxN* (aka *toxN*$_{Ec}$)	Guegler and Laub, 2021
ML3344	DH5α pBAD33	Guegler and Laub, 2021
gCKG-407	DH5α pBAD33-*toxN(K111N)*	This study
gCKG-408	DH5α pBAD33-*toxN(K111R)*	This study
gCKG-410	DH5α pBAD33-*toxN(Y115H)*	This study
gCKG-409	DH5α pBAD33-*toxN(L118S)*	This study
ML4100	DH5α pBAD33-*toxN*$_{Pa}$	This study
ML4101	DH5α pBAD33-*toxN*$_{Kp}$	This study
ML4102	DH5α pBAD33-*toxN*$_{Ss}$	This study
ML4104	DH5α pBAD33-*toxN*$_{Vk}$	This study
ML4105	DH5α pBAD33-*toxN*$_{Ser.}$	This study
ML4106	DH5α pBAD33-*tenpN*$_{Ec}$	This study
ML3345	DH5α pEXT20-*toxI*	Guegler and Laub, 2021
ML1978	DH5α pEXT20	E. coli Genetic Stock Center, #12325
ML3814	TOP10 pEXT20-*tifA*$_{T2}$	Srikant, Guegler, and Laub, 2022

ML3815	TOP10 pEXT20-*tifA*$_{T4}$	Srikant, Guegler, and Laub, 2022
ML3816	TOP10 pEXT20-*tifA*$_{T6}$	Srikant, Guegler, and Laub, 2022
ML3817	DH5α pEXT20-*tifA*$_{RB69}$	Srikant, Guegler, and Laub, 2022
ML4173	DH5α pEXT20-*tifA*$_{RB69}$*(P53A T54A)*	This study
ML4174	DH5α pEXT20-*tifA*$_{RB69}$*(K55A R56A R59A)*	This study
ML4146	DH5α pEXT20-*tifA*$_{T4}$*(F41S)*	This study
ML4147	DH5α pEXT20-*tifA*$_{T4}$*(F41H)*	This study
ML4148	DH5α pEXT20-*tifA*$_{RB69}$*(H41F)*	This study
ML4149	DH5α pEXT20-*tifA*$_{RB69}$*(H41S)*	This study
ML4107	DH5α pEXT20-*tifA*$_{EcS1}$	This study
ML4108	DH5α pEXT20-*tifA*$_{vB\_EcoM\_005}$	This study
ML4109	DH5α pEXT20-*tifA*$_{vB\_EcoM\_VR7}$	This study
ML4110	DH5α pEXT20-*tifA*$_{JS98}$	This study
ML4111	DH5α pEXT20-*tifA*$_{PM2}$	This study
ML4112	DH5α pEXT20-*tifA*$_{vB\_PatP\_CB4}$	This study
ML3346	DH5α pBR322-*toxIN*	Guegler and Laub, 2021
ML4114	DH5α pBR322-*toxI-toxN(K111N)*	This study
ML4115	DH5α pBR322-*toxI-toxN(K111R)*	This study
ML4116	TOP10 pBR322-*toxI-toxN(Y115H)*	This study
ML4117	TOP10 pBR322-*toxI-toxN(L118S)*	This study
ML3349	DH5α pBR322-*toxI-toxN*-His_6	Guegler and Laub, 2021

ML3810	DH5α pKVS45-*tifA*$_{T4}$	Srikant, Guegler, and Laub, 2022
ML3821	TOP10 pKVS45-*tifA*$_{T4}$-FLAG	Srikant, Guegler, and Laub, 2022
ML4175	DH5α pKVS45-*tifA*$_{T4}$*(P53A T54A)*-FLAG	This study
ML4176	DH5α pKVS45-*tifA*$_{T4}$*(K55A R56A R59A)*-FLAG	This study
ML4150	DH5α pKVS45-*tifA*$_{T4}$*(F41S)*-FLAG	This study
ML4151	DH5α pKVS45-*tifA*$_{T4}$*(F41H)*-FLAG	This study
ML4160	DH5α pTXB1-*toxN*	This study
ML4161	DH5α pACYC-*tifA*$_{RB69}$	This study; parental vector is pACYC Duet
ML4180	DH5α pACYC Duet-*toxI*-*tifA*$_{RB69}$	
ML4162	DH5α pACYC-*tifA*$_{RB69}$-FLAG	
ML4166	DH5α pET-*tifA*$_{RB69}$-MBP-His$_6$	This study; parental vector from Addgene (Plasmid #37237)
ML4167	DH5α pET-*tifA*$_{RB69}$*(P53A T54A)*-MBP-His$_6$	This study; parental vector from Addgene (Plasmid #37237)
ML4168	DH5α pET-*tifA*$_{RB69}$*(K55A R56A R59A)*-MBP-His$_6$	This study; parental vector from Addgene (Plasmid #37237)
ML3347	TOP10 pBR322-*toxI-toxN(K55A)*	Guegler and Laub, 2021
ML4184	DH5α pBR322-*toxI-toxN(K55A)*-His$_6$	This study
ML4186	DH5α pEXT20-*tifA*$_{RB69}$-FLAG	This study
ML4191	DH5α pEXT20-*tifA*$_{RB69}$-MBP-His$_6$	This study
ML4187	DH5α pEXT20-*tifA*$_{vB\_PatP\_CB4}$-FLAG	This study
ML4194	DH5α pEXT20-*tifA*$_{vB\_EcoM\_005}$-FLAG	This study

ML4196	DH5α pBAD33-*toxN*-chitin binding domain	This study

Table S4. Plasmids

Plasmid	Description	Source
pBAD33	Arabinose inducible vector	Guzman et al., 1995
pBAD33-*toxN* (*toxN*$_{Ec}$)	Arabinose inducible ToxN expression	Guegler and Laub, 2021
pBAD33-*toxN(K111N)*	Arabinose inducible ToxN escape mutant expression	This study
pBAD33-*toxN(K111R)*	Arabinose inducible ToxN escape mutant expression	This study
pBAD33-*toxN(Y115H)*	Arabinose inducible ToxN escape mutant expression	This study
pBAD33-*toxN(L118S)*	Arabinose inducible ToxN escape mutant expression	This study
pBAD33-*toxN*-chitin binding domain	Arabinose inducible ToxN fusion protein expression	This study
pBAD33-*toxN*$_{Pa}$	Arabinose inducible ToxN expression	This study
pBAD33-*toxN*$_{Kp}$	Arabinose inducible ToxN expression	This study
pBAD33-*toxN*$_{Ss}$	Arabinose inducible ToxN expression	This study
pBAD33-*toxN*$_{Vk}$	Arabinose inducible ToxN expression	This study
pBAD33-*toxN*$_{Ser}$	Arabinose inducible ToxN expression	This study
pBAD33-*tenpN*$_{Ec}$	Arabinose inducible ToxN expression	This study
pEXT20	IPTG inducible vector	E. coli Genetic Stock Center, #12325
pEXT20-*toxI*	IPTG inducible *toxI* expression	Guegler and Laub, 2021
pEXT20-*tifA*$_{T2}$	IPTG inducible *tifA* expression	Srikant, Guegler, and Laub, 2022

pEXT20-*tifA*$_{T4}$	IPTG inducible *tifA* expression	Srikant, Guegler, and Laub, 2022
pEXT20-*tifA*$_{T6}$	IPTG inducible *tifA* expression	Srikant, Guegler, and Laub, 2022
pEXT20-*tifA*$_{RB69}$	IPTG inducible *tifA* expression	Srikant, Guegler, and Laub, 2022
pEXT20-*tifA*$_{RB69}$*(P53A T54A)*	IPTG inducible *tifA* expression	This study
pEXT20-*tifA*$_{RB69}$*(K55A R56A R59A)*	IPTG inducible *tifA* expression	This study
pEXT20-*tifA*$_{T4}$(F41S)	IPTG inducible *tifA* expression	This study
pEXT20-*tifA*$_{T4}$(F41H)	IPTG inducible *tifA* expression	This study
pEXT20-*tifA*$_{RB69}$(H41F)	IPTG inducible *tifA* expression	This study
pEXT20-*tifA*$_{RB69}$(H41S)	IPTG inducible *tifA* expression	This study
pEXT20-*tifA*$_{RB69}$-FLAG	IPTG inducible *tifA*-FLAG expression	This study
pEXT20-*tifA*$_{RB69}$-MBP-His$_6$	IPTG inducible *tifA*-MBP-His$_6$ expression	This study
pEXT20-*tifA*$_{EcS1}$	IPTG inducible *tifA* expression	This study
pEXT20-*tifA*$_{vB\_EcoM\_005}$	IPTG inducible *tifA* expression	This study
pEXT20-*tifA*$_{vB\_EcoM\_VR7}$	IPTG inducible *tifA* expression	This study
pEXT20-*tifA*$_{JS98}$	IPTG inducible *tifA* expression	This study
pEXT20-*tifA*$_{PM2}$	IPTG inducible *tifA* expression	This study
pEXT20-*tifA*$_{vB\_PatP\_CB4}$	IPTG inducible *tifA* expression	This study
pEXT20-*tifA*$_{vB\_PatP\_CB4}$-FLAG	IPTG inducible *tifA*-FLAG expression	This study
pEXT20-*tifA*$_{vB\_EcoM\_005}$-FLAG	IPTG inducible *tifA*-FLAG expression	This study

pBR322-*toxIN*	Full *toxIN* locus	Guegler and Laub, 2021
pBR322-*toxI*-*toxN*-His_6	*toxIN* locus with C-terminal His_6-tagged ToxN	Guegler and Laub, 2021
pBR322-*toxI*-*toxN(K111N)*	*toxIN* locus with TifA-insensitive ToxN	This study
pBR322-*toxI*-*toxN(K111R)*	*toxIN* locus with TifA-insensitive ToxN	This study
pBR322-*toxI*-*toxN(Y115H)*	*toxIN* locus with TifA-insensitive ToxN	This study
pBR322-*toxI*-*toxN(L118S)*	*toxIN* locus with TifA-insensitive ToxN	This study
pBR322-*toxI*-*toxN(K55A)*-His_6	*toxIN* locus with nuclease-inactive, His_6-tagged ToxN	This study
pKVS45-$tifA_{T4}$	aTc inducible TifA expression	This study
pKVS45-$tifA_{T4}$-FLAG	aTc inducible expression of C-terminal FLAG-tagged TifA	This study
pKVS45-$tifA_{T4}$*(P53A T54A)*-FLAG	aTc inducible expression of C-terminal FLAG-tagged TifA mutant	This study
pKVS45-$tifA_{T4}$*(K55A R56A R59A)*-FLAG	aTc inducible expression of C-terminal FLAG-tagged TifA mutant	This study
pKVS45-$tifA_{T4}$*(F41S)*-FLAG	aTc inducible expression of C-terminal FLAG-tagged TifA mutant	This study
pKVS45-$tifA_{T4}$*(F41H)*-FLAG	aTc inducible expression of C-terminal FLAG-tagged TifA mutant	This study
pTXB1-*toxN*	IPTG inducible ToxN (with C-term chitin binding domain) overexpression driven by T7 RNA polymerase	This study
pACYC-$tifA_{RB69}$	IPTG inducible TifA overexpression driven by T7 RNA polymerase	This study

pACYC-*tifA*$_{RB69}$-FLAG	IPTG inducible TifA-FLAG overexpression driven by T7 RNA polymerase	This study
pACYC Duet-*toxI*-*tifA*$_{RB69}$	IPTG inducible *toxI* and TifA overexpression driven by T7 RNA polymerase	This study
pET-*tifA*$_{RB69}$-MBP-His_6	IPTG inducible TifA-MBP-His_6 overexpression driven by T7 RNA polymerase	This study
pET-*tifA*$_{RB69}$*(P53A T54A)*-MBP-His_6	IPTG inducible TifA(P53A T54A)-MBP-His_6 overexpression driven by T7 RNA polymerase	This study
pET-*tifA*$_{RB69}$*(K55A R56A R59A)*-MBP-His_6	IPTG inducible TifA(K55A R56A R59A)-MBP-His_6 overexpression driven by T7 RNA polymerase	This study
pACYC Duet-*toxI*-*tifA*$_{RB69}$-FLAG	IPTG inducible *toxI* and TifA-FLAG overexpression driven by T7 RNA polymerase	This study
pACYC Duet-*toxI*-*tifA*$_{RB69}$ *(K55A R56A R59A)*	IPTG inducible *toxI* and TifA(K55A R56A R59A) overexpression driven by T7 RNA polymerase	This study

Table S5. Primers and Oligos

Number	Purpose	Sequence (5′→3′)
CKG-499	Construct pBR322-*toxI-toxN(Y115H)* (forward)	CATAAACAGTTACAATTTATTCGTGTAAATGAAGATAAAATATCTG
CKG-500	Construct pBR322-*toxI-toxN(Y115H)* (reverse)	TAGCATTCTTTTGTAAGGCGTATCAGGCATG
CKG-501	Construct pBR322-*toxI-toxN(L118S)* (forward)	AGCCAATTTATTCGTGTAAATGAAGATAAAATATCTGAAAAATCAAAACTATTAAG
CKG-502	Construct pBR322-*toxI-toxN(L118S)* (reverse)	CTGTTTATATAGCATTCTTTTGTAAGGCGTATCAGGC
CKG-545	Construct pBR322-*toxI-toxN(K111N)* (forward)	AACAGAATGCTATATAAACAGTTACAATTTATTCGTGTAAATGAAG
CKG-546	Construct pBR322-*toxI-toxN(K111N/K111R)* (reverse)	GTAAGGCGTATCAGGCATGCTATCCAGATC
CKG-547	Construct pBR322-*toxI-toxN(K111R)* (forward)	CGTAGAATGCTATATAAACAGTTACAATTTATTCGTGTAAATGAAG
CKG-597	Construct pEXT20-$tifA_{T4}$*(F41S)* (forward)	TCTGGTCGAGTGGAATTTATTAAGTTTGCTGTTCGCC
CKG-598	Construct pEXT20-$tifA_{T4}$*(F41S)* and pEXT20-$tifA_{T4}$*(F41H)* (reverse)	TGGAGGGCTTTCTTCTGCTGGAATATC
CKG-599	Construct pEXT20-$tifA_{T4}$*(F41H)* (forward)	CATGGTCGAGTGGAATTTATTAAGTTTGCTGTTCGCC
CKG-600	Construct pEXT20-$tifA_{RB69}$*(H41F)* (forward)	TTTGGCAAAGTGGACTTCGTTAAATTTGCTGTAC
CKG-601	Construct pEXT20-$tifA_{RB69}$*(H41F)* and pEXT20-$tifA_{RB69}$*(H41S)* (reverse)	TGGAAACTTTTCAGATGCTTCACAGTCCATC

CKG-602	Construct pEXT20-*tifA*$_{RB69}$*(H41S)* (forward)	TCTGGCAAAGTGGACTTCGTTAAATTTGCTGTAC
CKG-625	Construct pEXT20-*tifA*$_{RB69}$*(K55A R56A R59A)* (forward)	CGTCCGACTGCGGCGCAGATTG
CKG-626	Construct pEXT20-*tifA*$_{RB69}$*(K55A R56A R59A)* (reverse)	TACAGCAAATTTAACGAAGTCCACTTTGCCG
CKG-603	Construct pKVS45-*tifA*$_{T4}$*(P53A T54A)* (forward)	GCGGCAAAGAGGCAGATTCGCCAATGCAAAAG
CKG-604	Construct pKVS45-*tifA*$_{T4}$*(P53A T54A)* (reverse)	GCGAACAGCAAACTTAATAAATTCCACTCGACC
CKG-523	Construct pKVS45-*tifA*$_{T4}$*(R52A K55A R56A R59A)* (forward)	CGCAATCTGCGCCGCCGTAGGCGCAACAGCAAACTT AATAAATTCCACTCGACCAAATGG
CKG-524	Construct pKVS45-*tifA*$_{T4}$*(R52A K55A R56A R59A)* (reverse)	CAATGCAAAAGATACTTTCGTAAAATCGTCAAGTTA GAGAAAC
CKG-411	Construct pKVS45-*tifA*$_{T4}$*(R52A K55A R56A R59A)*-FLAG(forward)	TTACTTGTCATCGTCGTCCTTGTAGTCGCTTCCGCTT CCTTTTAAAATTTCTGCGTAATCACATGTTACAAACT GTTTC
CKG-404	Construct pKVS45-*tifA*$_{T4}$*(R52A K55A R56A R59A)*-FLAG (reverse)	TTATGCATAATCCGGAACATCATACGGATAGCTTCC GCTTCCTTTTAAAATTTCTGCGTAATCACATGTTACA AACTGTTTC
CKG-627	Construct pKVS45-*tifA*$_{T4}$*(K55A R56A R59A)* (forward)	CGCCCTACGGCGGCGCAGATTG
CKG-628	Construct pKVS45-*tifA*$_{T4}$*(K55A R56A R59A)* (reverse)	AACAGCAAACTTAATAAATTCCACTCGACCAAATGG
CKG-413	Construct pTXB1-*toxN* (forward)	GGTGGTCATATGAAATTTTACACAATATCAAGCAGT TACATAAAATACCTG
CKG-415	Construct pTXB1-*toxN* (reverse)	GGTGGTTGCTCTTCCGCAGCGATCGTCTATTTCCATG TCTTCAGGTTTTTACCG

CKG-416	PCR-amplify *toxI* for construction of pACYC-*toxI* (forward)	CCCTGTAGAAATAATTTTGTTTAACTTTAATCCTAGTTGTAAGCCCAAGCGAAGATCATG
CKG-139	PCR-amplify *toxI* for construction of pACYC-*toxI* (reverse)	TTGCTCAGCGGTGGCAGCAGCATTTTTCTCGTAAAAAAGGCGACTGTGTATAG
CKG-147	Linearize pACYC for construction of pACYC-*toxI* (forward)	ATTAAAGTTAAACAAAATTATTTCTACAGGGGAATTGTTATC
CKG-134	Linearize pACYC for construction of pACYC-*toxI* (reverse)	CTGCTGCCACCGCTGAGCAATAAC
CKG-450	PCR-amplify $tifA_{RB69}$ for construction of pACYC-$tifA_{RB69}$ (forward)	GTTTAACTTTAATAAGGAGATATACCATGTATTCAACTGTGTTTAAACCATCAACATACG
CKG-451	PCR-amplify $tifA_{RB69}$ for construction of pACYC-$tifA_{RB69}$ (reverse)	CGGTGGCAGCAGCCTAGGTTAATCATTTTAAACTTTTGCGAAATTTGCGTTTTTCTTGAC
CKG-423	Linearize pACYC for construction of pACYC-$tifA_{RB69}$ (forward)	GGTATATCTCCTTATTAAAGTTAAACAAAATTATTTCTACAGGGGAATTG
CKG-424	Linearize pACYC for construction of pACYC-$tifA_{RB69}$ (reverse)	GGTATATCTCCTTATTAAAGTTAAACAAAATTATTTCTACAGGGGAATTG
CKG-460	PCR-amplify *toxI* for construction of pACYCDuet-1-*toxI*-$tifA_{RB69}$ (reverse)	TTACTTTCTGTTCGACTTAAGCATTACATTTTTTCTCGTAAAAAAGGCGACTGTGTATAG
CKG-444	Linearize pACYCDuet-1 for construction of pACYCDuet-1-*toxI*-$tifA_{RB69}$ (reverse)	TAATGCTTAAGTCGAACAGAAAGTAATCGTATTGTACAC
CKG-458	PCR-amplify $tifA_{RB69}$ for construction of pACYCDuet-1-*toxI*-$tifA_{RB69}$ (forward)	AGAGAGCATATGTATTCAACTGTGTTTAAACCATCAACATACGAATTTGC
CKG-459	PCR-amplify $tifA_{RB69}$ for construction of pACYCDuet-1-*toxI*-$tifA_{RB69}$ (reverse)	AGAGAGCCTAGGTTAATTATTTTAAACTTTTGCGAAATTTGCGTTTTTCTTGACG

CKG-465	PCR-amplify *tifA*$_{RB69}$ for construction of pET-*tifA*$_{RB69}$-MBP-His$_6$ (forward)	TTTAAGAAGGAGATATAGTTCATGTATTCAACTGTG TTTAAACCATCAACATACGAATTT
CKG-466	PCR-amplify *tifA*$_{RB69}$ for construction of pET-*tifA*$_{RB69}$-MBP-His$_6$ (reverse)	GGATTGGAAGTAGAGGTTCTCTTTTAAACTTTTGCGA AATTTGCGTTTTTCTTGACG
CKG-441	Construct pACYC-*tifA*$_{RB69}$-FLAG and pEXT20-*tifA*$_{RB69}$-FLAG (reverse)	CTACTTGTCATCGTCGTCCTTGTAGTCGCTTCCGCTT CCTTTTAAACTTTTGCGAAATTTGCGTTTTTCTTGAC GAATC
CKG-575	Construct pACYC-*tifA*$_{RB69}$-FLAG (forward)	TAATTAACCTAGGCTGCTGCCACCGCTG
CKG-656	Construct pACYC-*toxI*-*tifA*$_{RB69}$ *(K55A R56A R59A)* (forward)	GCGGCGCAGATTGCGCAAGAAAAACGCAAATTTCGC AAAAGTTTAA
CKG-657	Construct pACYC-*toxI*-*tifA*$_{RB69}$ *(K55A R56A R59A)* (reverse)	AGTCGGACGTACAGCAAATTTAACGAAG
CKG-303	Construct pBR322-*toxI*-*toxN(K55A)*-His$_6$ (forward)	CATCATCACCATCACCATTAACCATTCTTTTTATGG AATTGAAGGTCCAAAATC
CKG-304	Construct pBR322-*toxI*-*toxN(K55A)*-His$_6$ (forward)	GCTTCCGCTTCCATCGTCTATTTC
CKG-661	Construct pBAD33-*toxN*-chitin binding domain (reverse)	CCTGCAGGTCGACTCATTGAAGCTGCCACAAGGCAG G
CKG-662	Construct pBAD33-*toxN*-chitin binding domain (forward)	ATTCAGGAGGGATTGAGCTCATGAAATTTTACACAA TATCAAGCAGTTACATAAAATACC
CKG-663	PCR amplify MBP-His$_6$ for construction of pEXT20-*tifA*$_{RB69}$-MBP-His$_6$ (forward)	AATTTCGCAAAAGTTTAAAAGAGAACCTCTACTTCC AATCCGGCTCTAGC

CKG-664	PCR amplify MBP-His_6 for construction of pEXT20-*tifA*$_{RB69}$-MBP-His_6 (reverse)	GCATGCCTGCAGGTCGACCTAATGGTGATGGTGATGGTGGCTACTG
CKG-624	PCR amplify pEXT20-*tifA*$_{RB69}$ for construction of pEXT20-*tifA*$_{RB69}$-MBP-His_6 (forward)	GGATTGGAAGTAGAGGTTCTCTTTTAAACTTTTGCGAAATTTGCGTTTTTCTTG
CKG-665	PCR amplify pEXT20-*tifA*$_{RB69}$ for construction of pEXT20-*tifA*$_{RB69}$-MBP-His_6 and EXT20-*tifA*$_{RB69}$-FLAG (reverse)	TAGGTCGACCTGCAGGCATGCAAG
CKG-666	Construct pEXT20-*tifA*$_{vB\_PatP\_CB4}$-FLAG and pEXT20-*tifA*$_{vB\_EcoM\_005}$-FLAG, forward	GGAAGCGGAAGCGACTACAAGGACGACGATGACAAGTAGTAGGTCGACCTGCAGGCATG
CKG-670	Construct pEXT20-*tifA*$_{vB\_EcoM\_005}$-FLAG, reverse	GAGCCCGTACACATTGCACTCATATTG
CKG-672	Construct pEXT20-*tifA*$_{vB\_PatP\_CB4}$-FLAG, reverse	GAGGTAATGTGAGTAGTCAGCCATAAACTGAGC
CKG-658	15-nucleotide 56FAM-labeled RNA oligo derived from *artJ* sequence for fluorescence polarization experiments	56FAM/rCrArCrCrArGrArArArUrArUrArUrU
CKG-659	30-nucleotide 56FAM-labeled RNA oligo derived from *artJ* sequence for fluorescence polarization experiments	56FAM/rUrArCrUrArCrGrCrArCrCrArGrArArArUrArUrArUrUrCrArGrGrArUrCrA
CKG-660	45-nucleotide 56FAM-labeled RNA oligo derived from *artJ* sequence for fluorescence polarization experiments	56FAM/rArArArArCrGrGrUrArCrUrArCrGrCrArCrCrArGrArArArUrArUrArUrUrCrArGrGrArUrCrArGrCrArCrCrCrGrG

CKG-674	30-nucleotide 56FAM-labeled DNA oligo derived from *artJ* sequence for fluorescence polarization experiments	56FAM/TACTACGCACCAGAAATATATTCAGGATCA
CKG-675	45-nucleotide 56FAM-labeled DNA oligo derived from *artJ* sequence for fluorescence polarization experiments	56FAM/AAAACGGTACTACGCACCAGAAATATATTCAGGATCAGCACCCGG
CKG-676	45-nucleotide 56FAM-labeled RNA oligo derived from *rrsA* sequence for fluorescence polarization experiments	56FAM/rCrUrGrUrCrGrUrCrArGrCrUrCrGrUrGrUrUrGrUrGrArArArUrGrUrUrGrGrGrUrUrArArGrUrCrCrCrGrCrArArC
CKG-677	45-nucleotide 56FAM-labeled RNA oligo derived from *rrsA* sequence for fluorescence polarization experiments	56FAM/rArGrArArUrUrCrCrArGrGrUrGrUrArGrCrGrGrUrGrArArArUrGrCrGrUrArGrArGrArUrCrUrGrGrArGrGrArArU
CKG-678	45-nucleotide 56FAM-labeled RNA oligo derived from *rrsC* sequence for fluorescence polarization experiments	56FAM/rArGrArUrGrArGrArArUrGrUrGrCrCrUrUrCrGrGrGrArArCrCrGrUrGrArGrArCrArGrGrUrGrCrUrGrCrArUrGrG
CKG-680	30-nucleotide DNA oligo complementary to CKG-674 for fluorescence polarization experiments	TGATCCTGAATATATTTCTGGTGCGTAGTA
CKG-681	45-nucleotide DNA oligo complementary to CKG-675 for fluorescence polarization experiments	CCGGGTGCTGATCCTGAATATATTTCTGGTGCGTAGTACCGTTTT
oSS-219	Construct pEXT20-*tifA*$_{PM2}$, forward	CACACAGGAAACAGAATTCGAGCTCATGAATATTTATATTATGTATTTAGATAG

oSS-220	Construct pEXT20-*tifA*$_{PM2}$, reverse	GAAGCTTGCATGCCTGCAGGTCGACTTAATCTCTTTCTAAGAGGGCCATC
oSS-221	Construct pEXT20-*tifA*$_{vB\_PatP\_CB4}$, forward	CACACAGGAAACAGAATTCGAGCTCATGCGTACTATTAAAACCTCAGTTATG
oSS-222	Construct pEXT20-*tifA*$_{vB\_PatP\_CB4}$, reverse	GAAGCTTGCATGCCTGCAGGTCGACTTAGAGGTAATGTGAGTAGTCAGC
oSS-229	Construct pEXT20-*tifA*$_{EcS1}$, forward	CACACAGGAAACAGAATTCGAGCTCATGAAAACTTATATTCATTACTACCCAGG
oSS-230	Construct pEXT20-*tifA*$_{EcS1}$, reverse	GAAGCTTGCATGCCTGCAGGTCGACTCATAGGTCCTCTTTTTCATATAATTC
oSS-231	Construct pEXT20-*tifA*$_{vB\_EcoM\_005}$, forward	CACACAGGAAACAGAATTCGAGCTCATGTTTGTCTTAACTCAATTCTCTAAAG
oSS-232	Construct pEXT20- *tifA*$_{vB\_EcoM\_005}$, reverse	GAAGCTTGCATGCCTGCAGGTCGACTTAGAGCCCGTACACATTGCAC
oSS-233	Construct pEXT20-*tifA*$_{vB\_EcoM\_VR7}$, forward	CACACAGGAAACAGAATTCGAGCTCATGATTATTCAAATTATGAAACCCG
oSS-234	Construct pEXT20- *tifA*$_{vB\_EcoM\_VR7}$, reverse	GAAGCTTGCATGCCTGCAGGTCGACTTATTTTACTTTAGCGTAATCTAAAGTCAC
oSS-235	Construct pEXT20-*tifA*$_{JS98}$, forward	CACACAGGAAACAGAATTCGAGCTCATGAATATCGTACTAATGAAACCAAC
oSS-236	Construct pEXT20- *tifA*$_{JS98}$, reverse	GAAGCTTGCATGCCTGCAGGTCGACTTATTTTAACGAATCTTCCAAATAAATTTTAC

Table S6. G-blocks

Number	Purpose	Sequence (5′→3′)
gbCKG-4	Construct pBAD33-*toxN*$_{Pa}$	GCACAACAACAGTTGCTAGTTCCGTAGTTGTAAGCC<br>TAACCGAAGTTCAGGTGATTTGCTACCTTTAAGTGC<br>AGCTAGAAATTCAGGTGATTTACTACCTTTAAGTAA<br>TAAAGTAAAAGAGGCGACTACATAGTCGCCTTTTTT<br>ACGAGAAAAATATGAAATTCTACACTATATCAAGCA<br>AATACATTGAATATCTAAAGGAATTTGACGACAAGG<br>TTCCCAATAGCGAAGATCCTACCTACCAAAATCCTA<br>AGGCTTTCATTGGCATAGTATTAGAGATCCAGGGAC<br>ATAAGTATCTAGCCCCTCTGACATCCCCAAAAAAGT<br>GGCATAATAATGTTAAAGAGTCATCTCTTAGCTGCTT<br>TAAGCTCCATGAAAACGGCGTGCCCGAAAATCAGCT<br>TGGATTGATTAACCTGAAGTTCATGATTCCAATAATC<br>GAAGCTGAAGTGTCCTTACTTGATTTGGGCAACATG<br>CCTAATACCCCTTACAAGAGAATGCTCTATAAGCAG<br>CTCCAGTTTATTCGTGCAAATTCTGATAAAATAGCTT<br>CAAAATCAGATACTTTACGAAACCTCGTATTACAAG<br>GAAAAATGCAAGGAACATGTAATTTTTCCCTGTTAG<br>AAGAAAAATATCGAGATTTCGGTAAAGAAGCTGAG<br>GATACGGAAGAAGGCGAGTAA
gbCKG-13	Construct pBAD33-*toxN*$_{Kp}$	CCCGTTTTTTTGGGCTAGCGAATTCGAGCTCATGAAG<br>TTTTACACGATTAGTTCTCGTTATATTGAATATCTGA<br>AGGGCTTCGAGGACAAAGTACCTAATAGCGAAGATC<br>CCACTTACCAAAATCCAAAAGCGTTTATCGGAATTG<br>TTTTGGAGATCCAGGGTCATAAATATCTGGCTCCGCT<br>GACCTCACCGAAGCCGTGGCACTCTACCATTAAGGA<br>GTCCAGTCCATCCTTTTTTAAGTTACACGAAAACGGC<br>GTGCCCGAAAATCAGTTAGGACTTATCAATTTGAAA<br>TTTATGATCCCAATCATTGAAGCCGAAGTAAGTTTGT<br>TGGATTTGAACGGCATGCCTAATACGAGCTATAAAC<br>GTATGTTGTACAAACAACTGCAGTTTATTCGTGTAA<br>ACTCGGACAAAATTGCAGAAAAATCAGAGCTGTTGC<br>GTAATCTTGTGTTGCAAGGGAAAATGCAAGGGACTT<br>GTAATTTCTCGTTGTTAGAGGAAAAGTACCGCGACT

		TCGGGAAGGAGGCGAATGACATGGAGGAGGGCGAG<br>TAAGTCGACCTGCAGGCATGCAAGCTTGGCTGTTTT<br>G
gbCKG-14	Construct pBAD33-*tenpN*$_{Ec}$	CCCGTTTTTTTGGGCTAGCGAATTCGAGCTCATGAGC<br>AAACAGGATTACATACAGTTAAGAACCCTTACAGAC<br>CAGTTTTATGCTGATAACAAAGGCCTACAAGAGGCT<br>CTCGACGGCAGTAATGATGGAAAAGTAAGAGGCTA<br>CGGAATCGTAGTTATTGATCTCAACGGATTGGTGTTC<br>GGGATACCCTTACGAAGCCATTTAAACCATAAATTT<br>GGATTCGTTTCGGAGCGATCAGAAGGTGTTAAGAAA<br>GGACTGGATTACACCAAAGCGTTGCTAATTAAGAAA<br>GAAGAGTATGTATCGCGAGCCTATAAGATACCAACT<br>CCTGAATTTACCCATATCAATGATAACAAAGAAAAA<br>ATACAAGAAGATTTCAACAAATTCGTAAACAGATAC<br>ATAGAAGCGAACGTCAAGAAAGATGAAAACATACT<br>CAGAAACTATCGTTACTCTACGCTGAAAAACTATCA<br>CAAAGAATTAGGTTTAGAAGACTAAGTCGACCTGCA<br>GGCATGCAAGCTTGGCTGTTTTG
gbCKG-18	Construct pBAD33-*toxN*$_{Ss}$	CCCGTTTTTTTGGGCTAGCGAATTCGAGCTCATGAAA<br>TTTTATACGATTTCCAGTTCCTACATTAAATATCTGA<br>AAGACTTTGACGACAAAGTACCCAATTCAGAGGATC<br>CTACCTACAATAATCCTAAGGCTTTTATCGGAATTGT<br>CTTAGAAATTGAAGGCCACAAATATTTGGCTCCCCT<br>GACAAGCCCTAAGGCTTGGCACGCAAACATCAAGG<br>AGTCGAGCCCGGCCTTTTTCAAGCTGCATGAAAATG<br>GGGTTCCCGACAATCAACTTGGGCTGATTAACTTGA<br>AATTTATGATTCCCATCATTGAAGCCGAGGTCTCTCT<br>GTTAGACCTTGACAGCATGCCCGACACGCCATATAA<br>ACGTATGCTGTATAAACAATTGCAATTTATCCGCGTT<br>AATGAGGATAAGATCTCGGAAAAGTCCAAGTTACTG<br>CGTAACCTGGCCTTACAGGGGCGTATGCAGGGCACA<br>TGCGATTTTTCTGTGCTTGAGGAGAAGTACCAACATT<br>TTGGCAAGAAACCTGAAGATATGGAGATTGATGACT<br>AAGTCGACCTGCAGGCATGCAAGCTTGGCTGTTTTG

gbCKG-22	Construct pBAD33-$toxN_{Vk}$	CCCGTTTTTTTGGGCTAGCGAATTCGAGCTCATGAAG<br>TTTTACGTCGTCTCGAACGATTACATTAACCATTTGA<br>AGAAAGTAGACGCTCGCGTTCCTGATAATTATGATG<br>AACGCCGCGCATATGTAGGCGTCGTGATGGAGGTTT<br>GTGGGATTAAATATCTGGTGCCTTTGACAAGCCATA<br>AAACAAAACACAAAGATATTAAACCAGGTGCACAG<br>ACCGTATTTAAAATTCACGAATTGAATAACGAAGCG<br>AATCCCCTTGGTATGGCACAAATTAGCAATATGCTG<br>CCAGTTTTGGACTCTGAAATCCAGTTACTGGATATG<br>AAGGTACAGTCCGAAAATAAGAAGAAGCTGCTTAA<br>CTTACAGCAGCAGTTCTTACGTAAAAACTCGGAACG<br>TTTTGTCAAGAAAGCAATGCGTCTTTACGAATTGGT<br>AACGGTCAAGAAGGTGCCCGGTTTGGTTAAAAATTG<br>TTGTGACTTTAAAGCGCTTGAAGCCGCCCGCGCTGC<br>TTACATTCCGGCAAACCAGCGTCAGGCCTCTAGTGA<br>AGGTTTAGCGGCCCTTGCTGAGAAGTTTAACAGTTA<br>AGTCGACCTGCAGGCATGCAAGCTTGGCTGTTTTG
gbCKG-25	Construct pBAD33-$toxN_{Ser.}$	CCCGTTTTTTTGGGCTAGCGAATTCGAGCTCATGAAG<br>TTCTATGTGATCGCAGATAGTTATATCAATCATTTGG<br>TTGCGTGCGATCAGCACGTTTATAAGAATAAGGGAA<br>CACGCCCCTATATCGGTGTCGTCCTGGAGGTTAATG<br>GTGTAGAATTTCTTGCACCGCTTACCTCTTATAAAGA<br>GAAGCAAGACAAGATTCCCAATTCTTCACCGTTAAT<br>CTTTAAGATGTATGAGTTAGGTAATGAGGAGAACAA<br>ACTGGGCATGGTCCAAGTCAACAACATGGTACCCGT<br>ACTGAGCAGTGAGGTTGAACTTCTTGATTTGTCTACT<br>CAAGACGCCAAATACCAAAATTTGTTAAACATGCAG<br>CAACAGTTCCTTCGTAAAAATCAGGAGGAACTTCAA<br>AAGAAGGCCTCTAAATTGTATAAGATCGTCAGTCAA<br>GGCGTAGCGACCGGGATTGTTAATGTCTGCTGCGAC<br>TTTAAGGCGTTGGAGGCCGCAATGAAGACTTACGTT<br>CCGCCTGTCGCACAGGAGTCTGCTGTACCCACTGTA<br>GCGCAAGAAGCATCCAGCGAACCTCAGTCAGACCTG<br>CAAGATAAACTGTCCGCTCTGTGTGGTAAGTACAGT<br>AAGCGTTAAGTCGACCTGCAGGCATGCAAGCTTGGC<br>TGTTTTG

gbSS005	Construct pEXT20-*tifA*$_{PM2}$	CTAGGTTTCTCCATACAGGAGGTACCCATGAATATTT<br>ATATTATGTATTTAGATAGACCACACAGACCGCCCT<br>ATAAAAGTAATATGGGTTGGCAGGTTTATATTGAAA<br>CTCCTTGTAGTGATAGTTTGCCGTGGGGTTTAGTTGA<br>TGTGTTTAATTACTCTACAAAGCCAACAAAGAGGCA<br>AATTCGTAAGGCTAAAAAGGCTTTTTATAAAAATAT<br>AAAAAGCGAAATAGAATCTAGAAAAATGATGATGG<br>CCCTCTTAGAAAGAGATTAAGGGGATCCTCTAGAGT<br>CGACCTGCAGG
gbSS007	Construct pEXT20-*tifA*$_{EcS1}$	ACCTAGGTTTCTCCATACAGGAGGTACCCATGAAAA<br>CTTATATTCATTACTACCCAGGCTTTGTATACGCAAA<br>AGGCACTAAAGCCGAGTATAGTTTCAAAGCCTCCTT<br>TGAACTCATTATTGACCAAGATGCATCTGATAAGTT<br>ACGATTCGGTTGTGTTTCAGCTAAAGTTTTTGTAACT<br>CGCCCGTCCAAACGTCAAATTCGTCGTCAACAAAAA<br>GAATTTCGTAAAGAAATGAAAGCTCTTGAATTATAT<br>GAAAAAGAGGACCTATGAGGGGATCCTCTAGAGTC<br>GACCTGCAGGC
gbSS010	Construct pEXT20-*tifA*$_{vB\_EcoM\_005}$	GATACTGAGCACCTAGGTTTCTCCATACAGGAGGTA<br>CCCATGTTTGTCTTAACTCAATTCTCTAAAGCAGTGT<br>ATAGTTACTCTTGTCAATGGGAAGTCTTTATCGATTC<br>TCCTTCACCTAAGTACGGTGTTTTTGGACGAGTAACT<br>ACTCAAGCTTATGTTGCACGGCCTACTAAGCGTCAG<br>ATTCGTAAACTTAAAAAGGCTCATCGCCAACTGATT<br>AAAAGAAGGGAACAATATGAGTGCAATGTGTACGG<br>GCTCTAAGGGGATCCTCTAGAGTCGACCTGCAGGCA<br>TGCAAGCTTG
gbSS011	Construct pEXT20-*tifA*$_{vB\_EcoM\_VR7}$	AGGTTTCTCCATACAGGAGGTACCCATGATTATTCA<br>AATTATGAAACCCGTAAAAAGTGGTTTTTGGGACAA<br>GTCTTTTGGTAAGCACAAGTTTTATGCAGACCGCGT<br>AAACAGTCGTTGGGAACTTATTATTTCTCAACAAAC<br>CAAGGGACAAATGGACTTTGTAGAATTCTATGAATT<br>TGATGTTCGTCCAACTAAGCGTCAGATTCGCCAATG<br>CAAAAAGGCATTTCGCGTGTATGTCCGTGCATCAGT<br>CCTCCGTCGTCAACGTCAATTGGCAATGGAACCGCA

		GTTAGTGACTTTAGATTACGCTAAAGTAAAATAAGG GGATCCTCTAGAGTCGACCTGCA
gbSS012	Construct pEXT20-*tifA*$_{JS98}$	CACCTAGGTTTCTCCATACAGGAGGTACCCATGAAT ATCGTACTAATGAAACCAACTCCTAGTTCTTTTTGGA ATAAATCTTTCGGTAAGCATAATATGTTTATCCAACG CACCGTAAGCAGCTGGGAACTAGTTCTTTCAGTAGG TCATAACGATTATGAACAGGAATATGTAGAGTTCTT CGAATTTCCTGTGAAGCCTACTAAGCGTCAGATTCG TCAGACCAAACGTAAATTCCGTAAAATTTATTTGGA AGATTCGTTAAAATAAGGGGATCCTCTAGAGTCGAC CTGCAGGCAT
gbSS015	Construct pEXT20-*tifA*$_{vB\_PatP\_CB4}$	AGGTTTCTCCATACAGGAGGTACCCATGCGTACTAT TAAAACCTCAGTTATGTTCTTCCCTGCTGACCCTGTT GTAGTTACCAAAACCAAGCGTGATTGGATGTTAGCT GATATGGCTAGTCCTGCTCAGTGGAGCATAGCAGTA GATTTTATAGTTGATGCTAATCCCATGTCTGCATCCA TACACCAGTATGCCTTTGTAGCTAAGCCTACATCTAA ACAGGTGCGCCAGTGTAAGCGTAAAGCTGTCATTGA GCATGAACGTGAATTGGCTCAGTTTATGGCTGACTA CTCACATTACCTCTAAGGGGATCCTCTAGAGTCGAC CTGCA
